# Cross-Sectional Associations between Clinical Biochemistry and Nutritional Biomarkers and Sarcopenic Indices of Skeletal Muscle in the Baltimore Longitudinal Study of Aging

**DOI:** 10.1016/j.tjnut.2025.03.006

**Published:** 2025-03-08

**Authors:** Jamie Scott, Max Yates, Toshiko Tanaka, Luigi Ferrucci, Donnie Cameron, Ailsa A Welch

**Affiliations:** 1 Norwich Medical School, University of East Anglia, Norwich, United Kingdom; 2 Centre for Population Health Research, Faculty of Medicine and Health Sciences, University of East Anglia, Norwich, United Kingdom; 3 Norwich Epidemiology Centre, Faculty of Medicine and Health Sciences, Population Health, University of East Anglia, Norwich, United Kingdom; 4 Department of Rheumatology, Norfolk and Norwich University Hospital, Norwich, United Kingdom; 5 Translational Gerontology Branch, Intramural Research Program, National Institute on Aging, National Institutes of Health, Baltimore, MD, United States; 6 Department of Medical Imaging, Radboud University Medical Center, Nijmegen, The Netherlands

**Keywords:** sarcopenia, clinical biochemistry, nutritional biomarkers, nutrition, skeletal muscle, muscle mass, muscle strength, grip strength, physical performance, aging

## Abstract

**Background:**

Investigating relationships between nutritional and clinical biochemistry biomarkers and skeletal muscle mass, strength and function (sarcopenic indices) may *1*) highlight micronutrients of interest for potential preventive or treatment strategies for sarcopenia, or *2*) highlight biomarkers that may be useful for identifying individuals at risk of sarcopenia.

**Objectives:**

Investigate associations between nutritional biomarkers (vitamin D, vitamin B_12_, folate, magnesium, potassium, calcium, and iron), clinical biomarkers (hemoglobin, ferritin, albumin, creatinine, and hemoglobin A1c: HbA1c), and sarcopenic indices (appendicular lean mass: ALM); height-adjusted ALM: ALM_ht_; fat-free mass as a percentage of total body weight; extended short physical performance battery score: _ext_SPPB; height-adjusted hand grip strength: HGS_ht_; height-adjusted knee extension concentric strength, and; height-adjusted knee extension isometric strength) in males and females.

**Methods:**

Using multivariable linear regression analysis, we investigated cross-sectional associations between biomarkers and sarcopenic indices in data collected from 1761 participants (age 22–103 y) from the Baltimore Longitudinal Study of Aging.

**Results:**

Hemoglobin was positively associated with ALM (*β* = 0.20, *P* = 0.021), HGS_ht_ (*β* = 0.25, *P* = 0.001), and _ext_SPPB (*β* = 0.13, *P* = 0.024) in males, and with _ext_SPPB in females (*β* = 0.15, *P* = 0.019). In males, serum iron was positively associated with ALM_ht_ (*β* = 0.0021, *P* = 0.038) and _ext_SPPB (*β* = 0.0043, *P* = 0.045). In females, ferritin was positively associated with knee-extension strength measurements. Serum creatinine was positively associated with lean mass measures in males and females and with muscle strength and function measures in males with normal renal function (estimated glomerular filtration rate ≥60 mL/min/1.73 m^2^). In males, high HbA1c was associated with lower ALM_ht_ (*β* = –0.21, *P* = 0.023), _ext_SPPB (*β* = –0.40, *P* = 0.027), and HGS_ht_ (*β* = –0.56, *P* = 0.031). In males and females, magnesium was positively associated with _ext_SPPB, and potassium was positively associated with measures of knee-extension strength.

**Conclusions:**

The associations found between measures of iron status and creatinine and sarcopenic indices, in males in particular, indicate potential importance for muscle health. Future longitudinal and intervention studies are warranted to confirm these findings.

## Introduction

Skeletal muscle is important for posture, balance, and physical movement, but it also plays a major, often under-appreciated role in whole-body energy, protein, and glucose metabolism [[1], [2], [3]]. As we age, skeletal muscle mass, strength, and physical function naturally decline and may lead to the development of sarcopenia: the age-related loss of muscle mass, strength, and physical function [4]. The decline in muscle mass and strength generally occurs more rapidly in males than females [5,6]. Sarcopenia can increase the risk of obesity and type 2 diabetes [1,7], impair the ability to recover from acute injury or illness [1], has been linked to falls, frailty, morbidity, and mortality, and poses major challenges for healthcare systems [8]. The global prevalence of sarcopenia is estimated to range from 10% to 27% in adults [4], and because of an aging population, it is predicted to more than double in the next few decades [8]. There is an urgent need to find ways to identify people at risk of sarcopenia, and to develop effective preventive and treatment strategies.

Interest in finding susceptibility/risk or diagnostic biomarkers for sarcopenia has grown in recent years. Because of the multifactorial mechanisms implicated in the development of sarcopenia, it has been suggested that a single risk or diagnostic biomarker likely does not exist, and that a panel of biomarkers, related to the range of underlying mechanisms, may be required [[9], [10], [11]]*.* Several routinely collected clinical biomarkers—for example, albumin and hemoglobin—have been investigated as potential candidates [12], whereas other clinical biomarkers that are relevant to skeletal muscle health have received less attention, and have been rarely investigated concurrently within the same population. Malnutrition is a key risk factor for sarcopenia [13], but previous research on nutritional strategies for sarcopenia has mostly focused on dietary protein. Many micronutrients are also essential for optimal muscle health, having mechanistic actions, and are worthy of further investigation [14], but there has been limited research investigating associations between nutritional biomarkers and sarcopenia or its indices (muscle mass, strength, and function). Exploring associations between routinely collected and readily available nutritional and clinical biochemistry biomarkers and sarcopenic indices may highlight biomarkers that, in future, could be easily integrated into clinical practice as susceptibility/risk or diagnostic biomarkers. In addition, this may highlight micronutrients of interest for further research as potential preventive or treatment strategies.

Much of the research exploring potential biomarkers for sarcopenia has focused on their associations with sarcopenia diagnosis, but the apparent prevalence of sarcopenia can vary substantially depending on the diagnostic criteria used [4]. Investigating associations with sarcopenic indices may offer an alternative that reduces or removes potential variability in sarcopenia diagnosis. Additionally, exploring these associations in a healthy population of adults, largely free of sarcopenia, may highlight potential “risk” biomarkers for sarcopenia: biomarkers that are associated with declining muscle mass, strength, and function before sarcopenia develops. To date, limited research has explored associations between 1 or more clinical or nutritional biomarkers and sarcopenic indices: few studies include all 3 sarcopenic indices, and many investigate associations in males and females together. Investigating associations in males and females separately is important to account for gender differences in muscle mass, muscle strength, and the effect of aging on skeletal muscle loss [15].

To address limitations in previous research, the purpose of this study is to explore associations between a range of nutritional biomarkers (vitamin D, vitamin B_12_, folate, magnesium, calcium, potassium, and iron), clinical biomarkers (albumin, hemoglobin, ferritin, creatinine, and hemoglobin A1c: HbA1c), and measures of lean mass (appendicular lean mass: ALM; height-adjusted ALM: ALM_ht__;_ fat-free mass as a percentage of total body weight: FFM%), muscle strength (height-adjusted hand grip strength: HGS_ht__;_ height-adjusted knee extension concentric strength: KEC_ht__;_ and height-adjusted knee extension isometric strength: KEI_ht_) and muscle function (extended short physical performance battery score: _ext_SPPB) in males and females separately using cross-sectional data from the Baltimore Longitudinal Study of Aging (BLSA). These specific nutritional and clinical biomarkers were chosen as they have either previously been investigated in relation to sarcopenia or one of its indices, or they have known relevance for skeletal muscle physiology.

## Methods

### Study population

The BLSA is a study of normative human aging that commenced in 1958 [16]. The study recruits healthy volunteers aged ≥20 y, recruited primarily from residents in the vicinity of Baltimore, Maryland, who have no history of chronic disease (with the exception of controlled hypertension), cancer, musculoskeletal or neurological conditions, and who are free from physical or cognitive impairments at the time of enrollment. Study visits take place approximately every 4 y for younger adults (<60 y), every 2 y for adults aged 60–79 y, and once per year for adults aged ≥80 y. For this study, the most recent visit where each participant had a blood sample collected and clinical and nutritional biomarkers measured was selected. This provided cross-sectional data for 1761 participants aged between 22 and 103 y, collected between April 2003 and September 2021.

This study was granted ethical approval by the Institutional Review Board of the National Institute of Health. Participants provided informed consent at each study visit and were fully informed of the study procedures and any potential risks, and informed consent was received from all participants, in accordance with the Declaration of Helsinki.

### Blood measurements

Blood samples were collected from participants between 07:00 and 08:00 after a 12-h overnight fast. Hemoglobin was measured on a Sysmex XE-2100 hematology analyzer using an SLS detection method (Sysmex Corporation). HbA1c was measured on a Bio-Rad DiaSTAT Analyser using liquid chromatography (Bio-Rad Laboratories Inc.). Serum ferritin was measured on an ADVIA Centaur System using a 2-stage antibody sandwich method (Bayer). Serum concentrations of albumin, creatinine, vitamin B_12_, folate, magnesium, calcium, potassium, and iron were measured using a Dimension Vista 1500 System (Siemens Healthcare Diagnostics). Colorimetric assays were used to measure albumin (with polychromatic endpoints) and creatinine, magnesium, calcium, and iron (with bichromatic endpoints). A chemiluminescence assay was used to measure vitamin B_12_ and folate. Potassium was measured using indirect potentiometry. Serum vitamin D was measured using a chemiluminescent assay on a DiaSorin LIAISON Analyser (DiaSorin Inc.). Plasma C-reactive protein (CRP) was measured using a particle-enhanced immunopholometric assay on a BN II System (Siemens Healthcare Diagnostics).

### Anthropometry and body composition

At each study visit, height (m) and weight (kg) were measured using standard protocols and used to calculate BMI as weight/height^2^. Body composition was assessed through whole-body dual-energy X-ray absorptiometry (DXA) scans using a Prodigy Scanner (General Electric, software version 10.51.006). DXA-measured ALM (kg), the sum of nonfat nonbone soft tissue in the arms and legs, was used as a surrogate marker of appendicular skeletal muscle mass. To scale for body size, ALM was adjusted for height by dividing by height^2^ (ALM_ht_). Both ALM and ALM_ht_ were included as outcome measures as these lean mass measures are used in the diagnosis of sarcopenia [[17], [18], [19], [20]]. Total body lean mass (kg) was also divided by total body weight (kg) and multiplied by 100 to calculate FFM%. DXA-measured fat mass (kg) in the arms and legs was summed to calculate appendicular fat mass (AFM) (kg).

### Skeletal muscle strength

Hand grip strength (HGS) (kg) was measured 3 times for each hand using a handheld Smedley Hand Dynamometer (Stoetling), and the maximum HGS for each side was recorded. Maximum HGS for each hand was summed and divided by 2 to calculate the average HGS. To scale for differences in HGS associated with body size, average HGS was adjusted for height by dividing by height^2^ (HGS_ht_). Knee extension (KE) strength was assessed via maximum concentric [21] and isometric [22] KE peak torque (Nm), measured using a Biodex Multi-Joint System-Pro isokinetic dynamometer with Advantage Software version 4X (Biodex Medical Systems Inc.). For both measures, the dynamometer was placed on the tibia, peak torque was measured 3 times in each leg, and the maximum was recorded. Participants were instructed to apply maximum force to the dynamometer, moving at a constant speed of 30°/s for concentric measurements, and at a fixed knee flexion of 70° for isometric measurements. Peak torque measurements for each leg were summed and divided by 2 to calculate the average KE concentric and isometric strength (KEC and KEI, respectively). Both KEC and KEI were adjusted for height by dividing by height^2^ (KEC_ht_ and KEI_ht_, respectively).

### Skeletal muscle function

The _ext_SPPB was used to assess muscle function, as previously described by Simonsick et al. [23]. This test was developed to overcome the ceiling effect that is observed when the standard short physical performance battery is used in well-functioning older adult populations. Briefly, this battery assesses 3 standing balance positions (1 consisting of a single leg stance), 2 gait speed assessments, and the time taken to complete 5 chair stands. Scores are provided as a ratio and range from 0 (failure to complete all assessments) to 4 (achieves maximum scores for all assessments) [23].

### Physical activity

Physical activity was assessed using a standard questionnaire [24]. Participants reported the types, frequency, and duration of moderate and vigorous exercise activities they undertook over the previous 2 wk. This information was used to calculate the number of minutes spent engaging in high-intensity, vigorous physical activity, and 4 physical activity categories were created: “not active” (<30 min/wk); “moderately active” (30 to <75 min/wk); “active” (75 to <150 min/wk); and “highly active” (≥150 min/wk).

### Dietary supplements

Information on the use of dietary supplements was collected through self-reported semi-quantitative food frequency questionnaires (FFQ). The FFQ collected information on consumption of foods and drinks from 16 different food and beverage groups, and the frequency and duration of use of dietary supplements. The University of Minnesota Nutrient Data System for Research program was used to estimate participants’ nutrient intakes from foods alone, and from foods plus supplements. Intake of each micronutrient consumed from food was subtracted from the intake of each micronutrient consumed from food plus supplements to calculate intakes from supplements alone. Categorical variables for supplement use were created for all micronutrients. With the exception of potassium, these were categorized as “no supplement use,” “low supplement use,” “high supplement use,” or “missing supplement use information.” Potassium supplementation was categorized as “no supplementation,” “supplement use,” or “missing supplement information.” For further details, refer to the “Supplemental Methods.”

### Other covariates

Information on self-reported race was collected through a structured interview during study visits. Participants were grouped into 3 categories; the largest 2 groups were participants who self-identified as White or Black. The remaining racial groups—including American Indian or Alaska Native, Chinese, Filipino, Japanese, other Asian or Pacific Islander, multiracial, and those not classifiable—were merged into a single “other” category. Information on smoking status (never smoked, former smoker or current smoker), which was also assessed during the interview, was provided as smoking habit has adverse effects on skeletal muscle [25]. Poor renal function is associated with loss of muscle mass, strength, and physical function [26]; therefore, estimated glomerular filtration rate (eGFR) (mL/min/1.73m^2^) was calculated for participants using the Chronic Kidney Disease Epidemiology Collaboration (CKD-EPI) [27] equation:

$$\text{eGFR}=142\times \min\;{\left(\text{Scr}/k,1\right)}^{a}\times \max\;{\left(\text{Scr}/k,1\right)}^{–1.2}\times 0.9938^{\text{age}}\times 1.012\left[\text{if}\;\text{female}\right]$$

where *Scr* = serum creatinine, *k* = 0.9 for males and 0.7 for females, and *a* = –0.302 for males and –0.241 for females.

### Statistical analysis

Stata statistical software version 17 (Stata Corp.) was used for all statistical analyses. The Shapiro-Wilk test was used to assess the normality of continuous variables. Differences between males and females were investigated using Mann-Whitney *U* tests and Chi-squared tests for proportions. Correlations among lean mass, muscle strength, and muscle function measures were investigated using Spearman’s rho correlation coefficients. Univariate and multivariable linear regression analyses were completed to evaluate the association of each independent variable (hemoglobin, ferritin, albumin, creatinine, HbA1c, vitamin D, vitamin B_12_, folate, magnesium, potassium, calcium, and iron) with different sarcopenia-related dependent variables (ALM, ALM_ht_, FFM%, _ext_SPPB, HGS_ht_, KEC_ht_, and KEI_ht_).

For most biomarkers, few participants had concentrations outside of the “normal” range based on laboratory methods (Supplemental Table 1), and so associations between normal compared with low/high concentrations could not be explored. Exceptions include *1*) HbA1c, where 21.1% of participants had HbA1c > 6%, *2*) vitamin D, where 35.2% of participants had below-normal vitamin D (<30 ng/mL), but only 0.8% of participants had concentrations reflecting deficiency (<10 ng/mL), and *3*) folate, where 76.5% of participants had above-normal serum folate (>14 ng/mL). To test for linear trends, each biomarker was categorized into quintiles—except for magnesium, which was categorized into quartiles due to ties—and the median value for each quantile was entered into regression models as a continuous variable. Where there was evidence of a linear trend, the raw biomarker concentrations were entered into the model as a continuous variable. Where there was no linear trend, biomarker quantiles were entered into regression models as a categorical variable, with the lowest quantile as the reference category. The raw continuous and categorical models were compared for all biomarkers, and for most biomarkers, there was an agreement between models (that is, significant associations were seen in both, with evidence of a linear trend, or no significant associations were found in either). Given the large number of biomarkers and outcomes included, results for continuous models are reported for conciseness. There were 2 biomarkers where there was some disagreement between models: HbA1c and folate. These biomarkers were therefore categorized for analysis as follows. HbA1c was categorized as ≤6% and >6%, reflecting a clinically relevant cut-off point [28]. As there is disagreement around cut-off points reflecting low and high serum folate, and because of the wide range of serum folate concentrations in this cohort, folate was categorized as quintiles (Qs) (Supplemental Table 2).

To meet the linear regression assumptions, _ext_SPPB was normalized using a Box-Cox transformation, separately for males and females. For clinical biomarkers, 2 nested regression models were fitted. For nutritional biomarkers, 3 nested regression models were fitted. For all independent variables, model 1 was unadjusted. Model 2 was adjusted for age (years), race (Caucasian, Black American, and Other), smoking status (never, former, and current), and physical activity (not active, moderately active, active, and highly active). All models (except for those containing creatinine) were additionally adjusted for eGFR, as declining renal function can also affect the concentrations of many clinical and nutritional biomarkers in the body [[29], [30], [31], [32], [33], [34], [35], [36]]. Models containing creatinine were not adjusted for eGFR because of collinearity between creatinine and eGFR. Models containing ferritin were additionally adjusted for CRP. Models containing ALM were additionally adjusted for AFM and height. All other dependent variables were additionally adjusted for BMI. For nutritional biomarkers, model 3 was adjusted for supplement use for that particular nutrient, entered as a categorical variable as described previously. For further details, see Supplemental Methods. A sensitivity analysis was completed to investigate associations between creatinine and muscle outcomes for individuals with normal eGFR (≥60 mL/min/1.73 m^2^) as renal function impacts circulating creatinine concentrations. To allow for graphical representation of results from models where biomarkers were included as continuous variables, and comparison of effect sizes between biomarkers and sarcopenic indices in males and females, all independent and dependent variables were standardized using Stata’s “std” function, and fully adjusted multivariable linear regression analysis was completed for each combination of standardized independent and dependent variables, as above, to provide standardized regression coefficients and 95% confidence intervals (CI).

**FIGURE 1:**
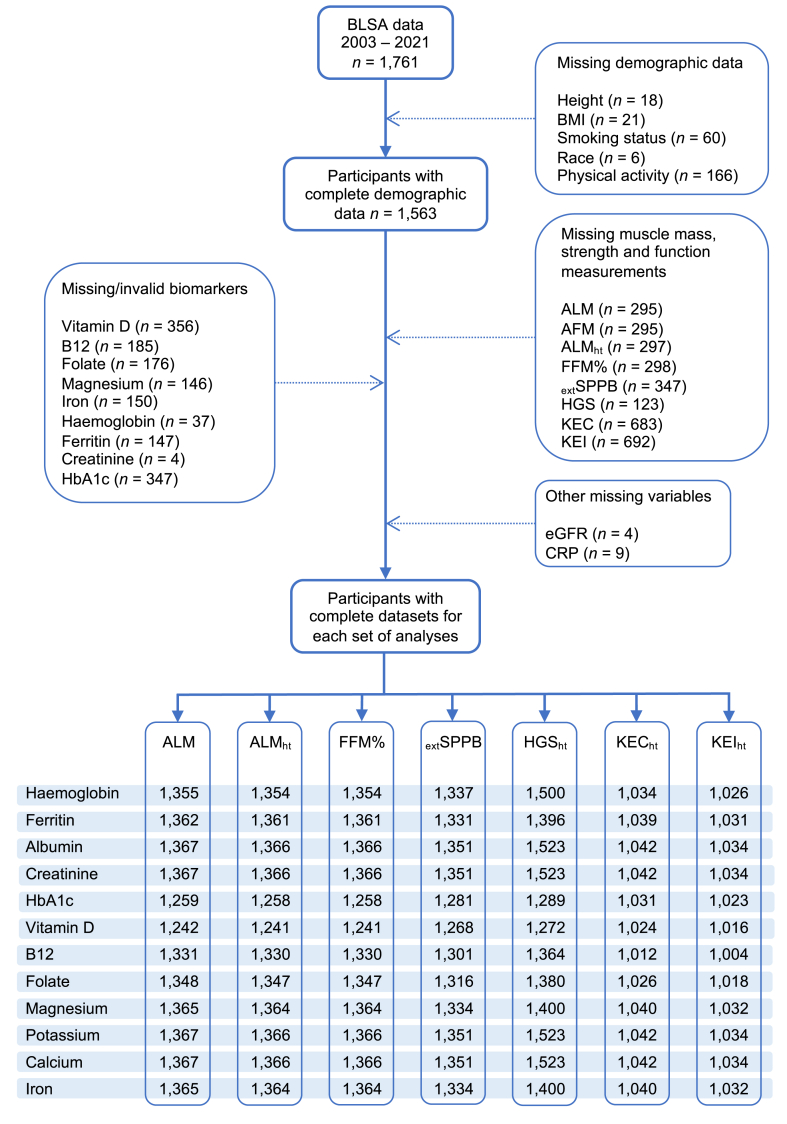
Flowchart of study participants with missing data, and the number of participants included in each set of regression analyses. AFM, appendicular fat mass; ALM, appendicular lean mass; ALM_ht_, height-adjusted ALM; BLSA, Baltimore Longitudinal Study of Aging; CRP, C-reactive protein; eGFR, estimated glomerular filtration rate; _ext_SPPB, extended short physical performance battery; FFM%, fat-free mass as a percentage of total body weight; HbA1c, hemoglobin A1c; HGS, hand grip strength; HGS_ht_, height-adjusted HGS; KEC, knee extension concentric strength; KEC_ht_, height-adjusted KEC; KEI, knee extension isometric strength; KEI_ht_, height-adjusted KEI.

To allow for graphical representation of results from models containing folate, adjusted means for each outcome measure were calculated for each quintile of serum folate via linear regression analysis. Where significant associations were found between biomarkers and muscle outcomes, we then conducted a sensitivity analysis in males and females aged ≥65 y to determine whether these associations were still present in older adults only. All analyses were stratified by gender. Figure 1 shows the number of participants with complete datasets for regression models. A *P* value <0.05 was considered statistically significant.

## Results

### Participant characteristics

Information on invalid and missing measurements can be found in Figure 1, with further information in the Supplemental Results. Table 1 contains demographic and clinical characteristics of the study population. The median age of participants was 74 (range 22–103) y, with females slightly younger than males: 72 (range 24–103) compared with 75 (range 22–99) y, respectively. Most participants (70.1%) were White American. Males were more likely to be current or former smokers (*P* = 0.017), have a “highly active” physical activity status (*P* < 0.001), and had higher lean mass and strength measurements compared with females (*P* for all <0.0001). With the exception of HbA1c, concentrations of clinical biochemistry biomarkers were higher in males (*P* for all <0.001). Females had higher serum vitamin D, vitamin B_12_, folate, and calcium (*P* for all <0.05), and lower serum potassium and iron than males (*P* for both <0.0001). Supplemental Table 3 shows the proportion of participants using dietary supplements.

**TABLE 1:** Participant characteristics, including demographic data, clinical biomarkers, serum levels of micronutrients, and measures of muscle mass, strength and function^1^.

	All	Females	Males	*P* value^2^
**Population characteristics**				
Age (y)	74 (61–83)	72 (60–82)	75 (63–84)	0.0021∗
	*n* = 1761	*n* = 891	*n* = 870	
Weight (kg)	75.3 (64.2–86.9)	66.9 (59.0–78.1)	82.0 (73.8–92.5)	<0.0001∗
	*n* = 1745	*n* = 887	*n* = 858	
Height (cm)	167.4 (160.5–174.6)	161.0 (156.9–165.4)	174.4 (169.5–179.3)	<0.0001∗
	*n* = 1743	*n* = 885	*n* = 858	
BMI (kg/m^2^)	26.4 (23.7–29.9)	25.8 (22.8–29.7)	26.9 (24.5–30.1)	<0.0001∗
	*n* = 1740	*n* = 885	*n* = 855	
Race, *n* (%)				<0.0001∗
White American	1230 (70.1)	582 (65.5)	648 (74.8)	
Black American	406 (23.1)	242 (27.3)	164 (18.9)	
Other	119 (6.8)	64 (7.2)	55 (6.3)	
Smoking status, *n* (%)				0.017∗
Never smoked	1059 (62.3)	564 (65.6)	495 (58.9)	
Former smoker	589 (34.6)	272 (31.6)	317 (37.7)	
Current smoker	53 (3.1)	24 (2.8)	29 (3.4)	
Physical activity, *n* (%)				<0.001∗
Not active	252 (15.8)	135 (16.3)	117 (15.2)	
Moderately active	627 (39.3)	350 (42.4)	277 (36.0)	
Active	383 (24.0)	205 (24.8)	178 (23.2)	
Highly active	333 (20.9)	136 (16.5)	197 (25.6)	
eGFR (mL/min/1.73 m^2^)	80.4 (66.5–91.6)	81.7 (68.2–92.7)	79.1 (64.9–90.6)	0.006∗
	*n* = 1757	*n* = 888	*n* = 869	
**Clinical biomarkers**				
Hemoglobin (g/dL)	13.4 (12.5–14.3)	13.0 (12.2–13.6)	14.0 (13.1–14.9)	<0.0001∗
	*n* = 1724	*n* = 874	*n* = 850	
Ferritin (ng/mL)	76.0 (42.8–126.0)	57.7 (35.4–97.4)	99.0 (59.2–157.2)	<0.0001∗
	*n* = 1614	*n* = 814	*n* = 800	
Albumin (g/dL)	3.8 (3.6–4.1)	3.8 (3.5–4.0)	3.9 (3.6–4.1)	0.0001∗
	*n* = 1761	*n* = 891	*n* = 870	
Creatinine (mg/dL)	0.90 (0.76–1.06)	0.79 (0.70–0.90)	1.00 (0.90–1.17)	<0.0001∗
	*n* = 1757	*n* = 888	*n* = 869	
HbA1c (%)	5.7 (5.4–6.0)	5.7 (5.4–6.0)	5.7 (5.4–6.0)	0.22
	*n* = 1414	*n* = 738	*n* = 676	
CRP (μg/mL)	1.01 (0.34–2.56)	1.15 (0.37–2.89)	0.90 (0.30–2.27)	0.0046∗
	*n* = 1752	*n* = 885	*n* = 867	
**Nutritional biomarkers**				
Vitamin D (ng/mL)	34.0 (26.8–42.0)	35.0 (28.0–44.0)	32.0 (25.0–40.0)	<0.0001∗
	*n* = 1405	*n* = 730	*n* = 675	
B_12_ (pg/mL)	560 (411–773)	583 (430–793)	536 (398–749)	0.0019∗
	*n* = 1576	*n* = 789	*n* = 787	
Folate (ng/mL)	19.5 (14.5–29.2)	20.0 (14.9–30.5)	19.1 (13.9–28.1)	0.002∗
	*n* = 1585	*n* = 802	*n* = 783	
Magnesium (mg/dL)	2.1 (2.0–2.2)	2.1 (2.0–2.2)	2.1 (1.9–2.2)	0.35
	*n* = 1615	*n* = 814	*n* = 801	
Potassium (mmol/L)	4.1 (3.9–4.3)	4.0 (3.9–4.2)	4.1 (3.9–4.3)	<0.0001∗
	*n* = 1761	*n* = 891	*n* = 870	
Calcium (mg/dL)	9.0 (8.7–9.3)	9.0 (8.7–9.3)	8.9 (8.7–9.3)	0.017∗
	*n* = 1761	*n* = 891	*n* = 870	
Iron (μg/dL)	83 (66–105)	80 (63–101)	86 (68–109)	<0.0001∗
	*n* = 1611	*n* = 814	*n* = 797	
**Body composition**				
AFM (kg)	11.4 (8.6–15.1)	13.4 (10.3–17.2)	9.6 (7.4–12.2)	<0.0001∗
	*n* = 1466	*n* = 754	*n* = 712	
ALM (kg)	20.2 (16.8–24.7)	17.1 (15.3–19.0)	24.7 (21.9–27.4)	<0.0001∗
	*n* = 1466	*n* = 754	*n* = 712	
ALM_ht_ (kg/m^2^)	7.2 (6.4–8.2)	6.5 (6.0–7.1)	8.1 (7.4–8.9)	<0.0001∗
	*n* = 1464	*n* = 754	*n* = 710	
FFM%	61.3 (54.8–67.5)	56.7 (51.2–62.6)	65.7 (60.8–71.5)	<0.0001∗
	*n* = 1463	*n* = 754	*n* = 709	
**Muscle strength and function**				
HGS (kg)	28 (21–36)	22 (18–28)	36 (28–44)	<0.0001∗
	*n* = 1638	*n* = 837	*n* = 801	
HGS_ht_ (kg/m^2^)	10.0 (7.9–12.4)	8.7 (7.0–10.3)	11.8 (9.4–14.0)	<0.0001∗
	*n* = 1627	*n* = 834	*n* = 793	
KEC (Nm)	96.7 (72.7–130.8)	81.3 (62.4–104.4)	122.9 (92.4–161.1)	<0.0001∗
	*n* = 1078	*n* = 571	*n* = 507	
KEC_ht_ (Nm/m^2^)	34.6 (26.9–44.8)	30.9 (24.7–38.9)	40.0 (30.9–51.0)	<0.0001∗
	*n* = 1076	*n* = 571	*n* = 505	
KEI (Nm)	108.8 (80.2–146.9)	91.3 (69.2–114.7)	138.1 (103.7–182.2)	<0.0001∗
	*n* = 1069	*n* = 566	*n* = 503	
KEI_ht_ (Nm/m^2^)	39.2 (30.0–50.3)	34.9 (27.2–43.4)	45.2 (34.3–57.2)	<0.0001∗
	*n* = 1067	*n* = 566	*n* = 501	
_ext_SPPB Score	2.64 (2.05–2.97)	2.64 (2.09–2.96)	2.64 (1.98–2.99)	0.99
	*n* = 1414	*n* = 739	*n* = 675	

Abbreviations: AFM, appendicular fat mass; ALM, appendicular lean mass; ALM_ht_, height-adjusted ALM; CRP, C-reactive protein; eGFR, estimated glomerular filtration rate; _ext_SPPB, extended short physical performance battery score; FFM%, fat-free mass as a percentage of total body weight; HbA1c, hemoglobin A1c; HGS, hand grip strength; HGS_ht_, height-adjusted HGS; KEC, knee extension concentric strength; KEC_ht_, height-adjusted KEC; KEI, knee extension isometric strength; KEI_ht_, height-adjusted KEI.

1 Data are expressed as median (IQR) or *n* (%).

∗ Statistically significant.

2 Differences between males and females were assessed using Mann-Whitney *U* tests. Differences for smoking status, race, and physical activity groups were assessed using Pearson chi-squared tests.

### Correlation between lean mass, muscle strength, and muscle function

Supplemental Figure 1 shows matrices of the correlations between sarcopenic indices in males and females, with further information in the Supplemental Results. Correlations between measures of muscle strength were strong (*r*_*s*_ ≥0.6) or very strong (*r*_*s*_ ≥0.9) and similar in both males and females. Weak (*r*_*s*_ < 0.4)-to-moderate (*r*_*s*_ = 0.4 to 0.59) correlations were found between the 3 types of sarcopenic indices (lean mass, muscle strength, and physical function), with generally stronger correlations in males than females. FFM% was not significantly correlated with any muscle strength measure in females, or with KEI_ht_ in males.

### Clinical biomarkers and lean mass

**FIGURE 2:**
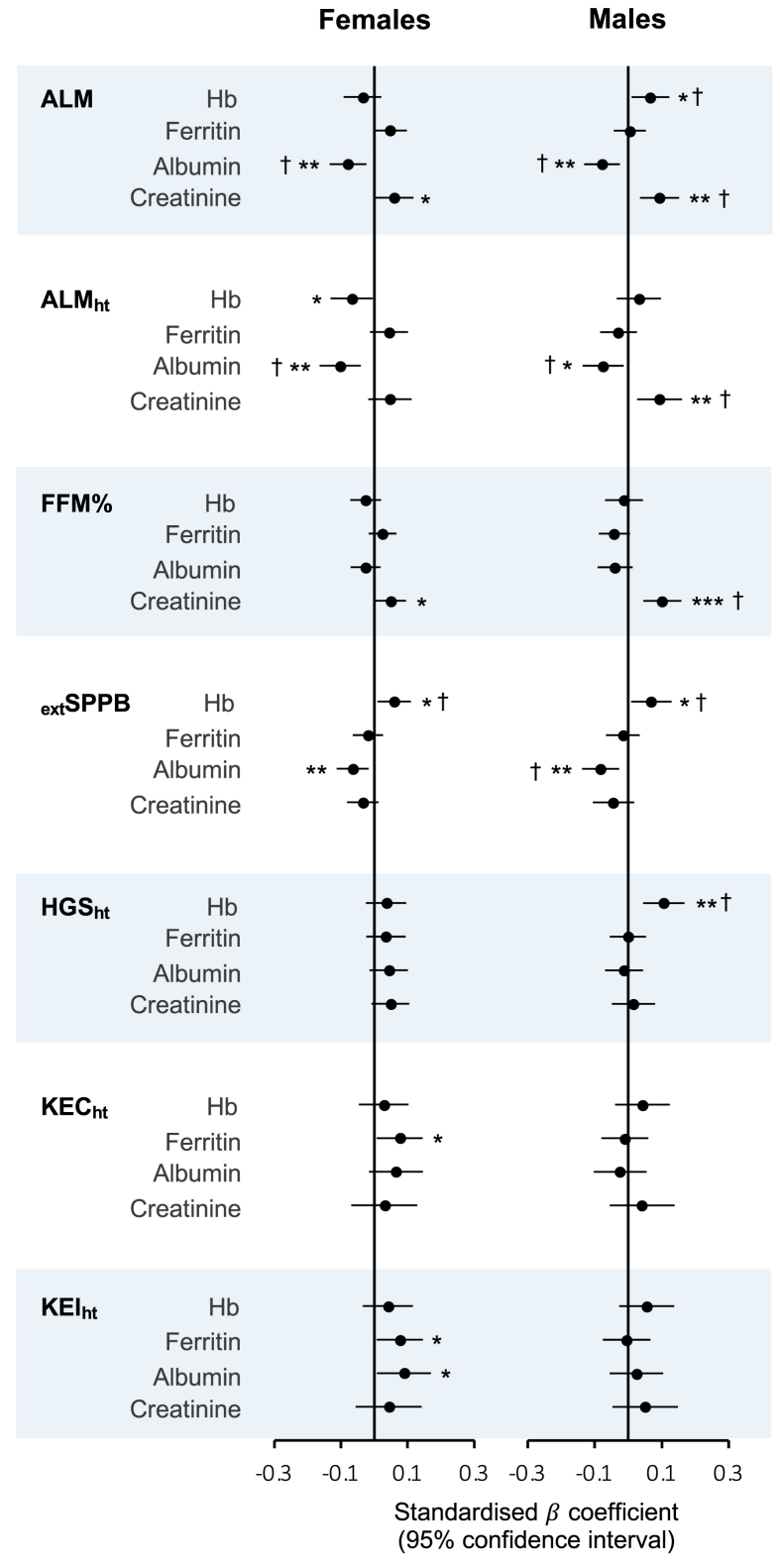
Forest plots of associations between clinical biomarkers and measures of lean mass, strength and function in females (left) and males (right). Data are presented as standardized β coefficient (95% confidence interval). Extended short physical performance battery score (_ext_SPPB) was transformed using a Box-Cox transformation before standardization. All models were adjusted for age (years), smoking status (never, former, current), race (White American, Black American, and Other), physical activity level (not active, moderately active, active, and highly active), and estimated glomerular filtration rate (mL/min/1.73 m^2^). Models containing creatinine were not adjusted for eGFR because of collinearity. Models containing ferritin were additionally adjusted for C-reactive protein category (≤5μ g/mL, >5 μ g/mL). Appendicular lean mass models (ALM) were additionally adjusted for appendicular fat mass (kg) and height (m). ALM adjusted for height (ALM_ht_), fat-free mass percent (FFM%), _ext_SPPB, height-adjusted grip strength (HGS_ht_), and height-adjusted knee extension concentric (KEC_ht_) and isometric (KEI_ht_) strength models were additionally adjusted for BMI (kg/m^2^). ∗*P* < 0.05, ∗∗*P* < 0.01, ∗∗∗*P* < 0.001. † indicates that a significant association was also found when models included older adults only (≥65 y).

Full results of associations between clinical biomarkers and lean mass outcomes can be found in Supplemental Tables 4 and 5, and forest plots of standardized regression coefficients (95% CI) for fully adjusted models in Figure 2. Results for HbA1c are presented separately in Table 2. In fully adjusted (nonstandardized) models, hemoglobin was positively associated with ALM in males (*β* = 0.20, *P* = 0.021) and negatively associated with ALM_ht_ in females (*β* = –0.057, *P* = 0.034). Higher albumin was negatively associated with both ALM (*β* = –0.94, *P* = 0.004; *β* = –0.64, *P* = 0.005) and ALM_ht_ (*β* = –0.24, *P* = 0.017; *β*=-0.26, *P* = 0.001) in males and females, respectively. In males only, high HbA1c (>6%) was associated with lower ALM_ht_ (*β* = –0.21, *P* = 0.023). A positive association was found between creatinine and all lean mass measures in males (ALM: *β* = 1.28, *P* = 0.002; ALM_ht_: *β* = 0.34, *P* = 0.006; FFM%: *β* = 2.64, *P* < 0.001) and with ALM (*β* = 0.78, *P* = 0.045) and FFM% (*β* = 1.79, *P* = 0.044) in females. In the sensitivity analysis (participants with normal eGFR), in males, the associations between creatinine and ALM (*β* = 3.27, *P* < 0.001) and ALM_ht_ (*β* = 0.97, *P* < 0.001) were strengthened, but the association with FFM% became nonsignificant. In females with normal eGFR, strong positive associations were observed between creatinine and lean mass measures (ALM: *β* = 3.26, *P* < 0.001; ALM_ht_: *β* = 1.07, *P* < 0.001; FFM%: *β* = 5.41, *P* = 0.002) (see Supplemental Figure 2).

**TABLE 2:** Associations between HbA1c and sarcopenic indices in females (top) and males (bottom)^1^

	HbA1c > 6% vs. ≤ 6%						
Females	Model 1^2^				Model 2^3^		
	*n*	*β ±* SE	*R*^2^	*P*	*β ±* SE	*R*^2^	*P*
ALM^4^ (kg)	665	0.37 (0.28)	0.003	0.20	0.36 (0.20)	0.54	0.08
ALM_ht_^5^ (kg/m^2^)	665	0.23 (0.09)	0.01	0.011∗	0.015 (0.073)	0.42	0.84
FFM%^5^	665	–3.61 (0.81)	0.03	<0.001∗	0.81 (0.48)	0.71	0.09
Extended SPPB score^5^^,^^6^	676	–1.29 (0.26)	0.04	<0.001∗	–0.12 (0.18)	0.61	0.49
HGS_ht_^5^ (kg/m^2^)	678	–0.11 (0.24)	0.0003	0.66	0.093 (0.21)	0.35	0.65
KEC_ht_^5^ (Nm/m^2^)	547	–1.84 (1.19)	0.004	0.12	0.52 (1.00)	0.39	0.60
KEI_ht_^5^ (Nm/m^2^)	542	–0.81 (1.33)	0.0007	0.54	1.14 (1.11)	0.39	0.31

Males	Model 1^2^				Model 2^3^		
	*n*	*β ±* SE	*R*^2^	*P*	*β ±* SE	*R*^2^	*P*
ALM^4^ (kg)	594	–0.61 (0.43)	0.003	0.16	–0.14 (0.29)	0.59	0.63
ALM_ht_^5^ (kg/m^2^)	593	–0.064 (0.12)	0.0005	0.58	–0.21 (0.09)	0.46	0.023∗
FFM%^5^	593	–3.97 (0.81)	0.04	<0.001∗	–0.48 (0.54)	0.62	0.38
Extended SPPB score^5^^,^^6^	605	–1.11 (0.25)	0.03	<0.001∗	–0.40 (0.18)	0.55	0.027∗
HGS_ht_^5^ (kg/m^2^)	611	–1.03 (0.32)	0.02	0.001∗	–0.56 (0.26)	0.44	0.031∗
KEC_ht_^5^ (Nm/m^2^)	484	–4.06 (1.65)	0.01	0.014∗	–2.24 (1.37)	0.42	0.10
KEI_ht_^5^ (Nm/m^2^)	481	–3.51 (1.88)	0.007	0.06	–2.60 (1.56)	0.42	0.10

Abbreviations: ALM, appendicular lean mass; ALM_ht_, height-adjusted ALM; FFM%, fat-free mass as a percentage of total body weight; HbA1c, hemoglobin A1c; HGS_ht_, height-adjusted hand grip strength; KEC_ht_, height-adjusted knee extension concentric strength; KEI_ht_, height-adjusted knee extension isometric strength; SPPB, short physical performance battery.

1 Data are presented as regression coefficients (β) ± SE, comparing HbA1c >6% to the reference category of HbA1c ≤6%.

2 Model 1 is unadjusted.

3 Model 2 is adjusted for age (years), smoking status (never, former or current), race (White American, Black American, Other) physical activity level (not active, moderately active, active, highly active), and estimated glomerular filtration rate (mL/min/1.73 m^2^) for all outcomes.

4 Models containing ALM are additionally adjusted for height (cm) and appendicular fat mass (kg).

5 ALM_ht_, FFM%, extended SPPB score, HGS_ht_, KEC_ht_, and KEI_ht_ models are additionally adjusted for BMI (kg/m^2^).

6 Extended SPPB score was transformed using a Box-Cox transformation in all models.

∗ Denotes statistical significance.

### Clinical biomarkers and muscle strength and function

In fully adjusted (nonstandardized) models, hemoglobin was positively associated with _ext_SPPB in both males (*β* = 0.13, *P* = 0.024) and females (*β* = 0.15, *P* = 0.019), and with HGS_ht_ in males (*β* = 0.25, *P* = 0.001). In females only, there were positive associations between ferritin and KEC_ht_ (*β* = 0.012, *P* = 0.031) and KEI_ht_ (*β* = 0.013, *P* = 0.031). Albumin was inversely associated with _ext_SPPB in males (*β* = –0.60, *P* = 0.003) and females (*β* = –0.50, *P* = 0.008); however, a positive association was found between albumin and KEI_ht_ in females (*β* = 2.89, *P* = 0.032). Higher HbA1c (>6%) was associated with lower _ext_SPPB (*β* = –0.40, *P* = 0.027) and lower HGS_ht_ (*β* = –0.56, *P* = 0.031) in males (Table 2, Supplemental Tables 6 and 7, Figure 2). Following sensitivity analyses (in participants with normal eGFR), creatinine was positively associated with all muscle strength and function measurements in males (_ext_SPPB: *β* = 1.66, *P* = 0.002; HGS_ht_: *β* = 1.55, *P* = 0.037; KEC_ht_: *β* = 9.86, *P* = 0.022; KEI_ht_: *β* = 12.68, *P* = 0.009), but not in females (see Supplemental Figure 2).

### Nutritional biomarkers and lean mass

**FIGURE 3:**
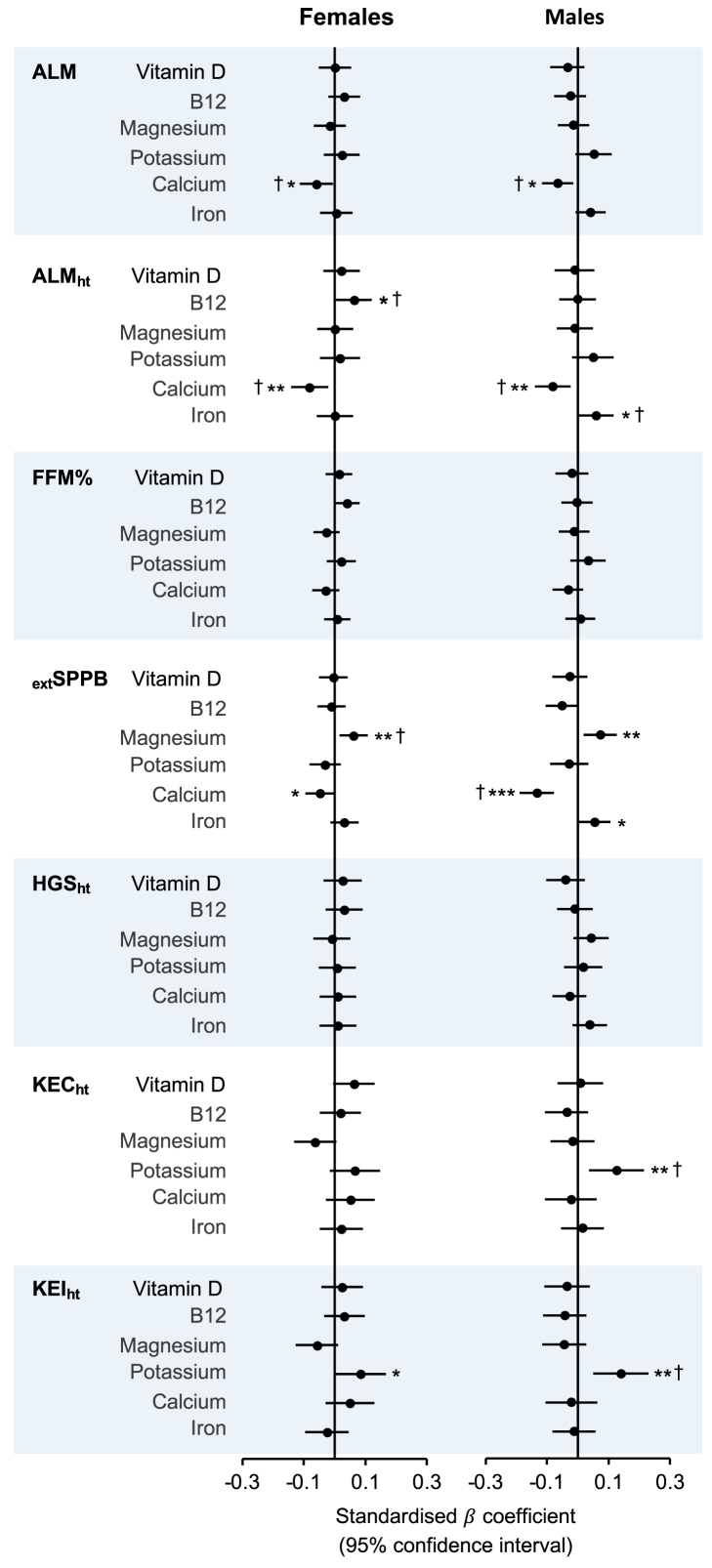
Forest plots of associations between nutritional biomarkers and measures of lean mass, strength and function in females (left) and males (right). Data are presented as standardized *β* coefficient (95% confidence interval). Extended short physical performance battery score (_ext_SPPB) was transformed using a Box-Cox transformation before standardization. All models were adjusted for age (years), smoking status (never, former, and current), race (White American, Black American, and Other), physical activity level (not active, moderately active, active, and highly active), estimated glomerular filtration rate (mL/min/1.73 m^2^) and use of dietary supplements for the corresponding nutrient as detailed in the Supplemental Methods. Appendicular lean mass (ALM) models were additionally adjusted for appendicular fat mass (kg) and height (m). ALM adjusted for height (ALM_ht_), fat-free mass percent (FFM%), _ext_SPPB, height-adjusted grip strength (HGS_ht_), and height-adjusted knee extension concentric (KEC_ht_) and isometric (KEI_ht_) strength models were additionally adjusted for BMI (kg/m^2^). ∗*P* < 0.05, ∗∗*P* < 0.01, ∗∗∗*P* < 0.001. † indicates that a significant association was also found when models included older adults only (≥65 y).

**FIGURE 4:**
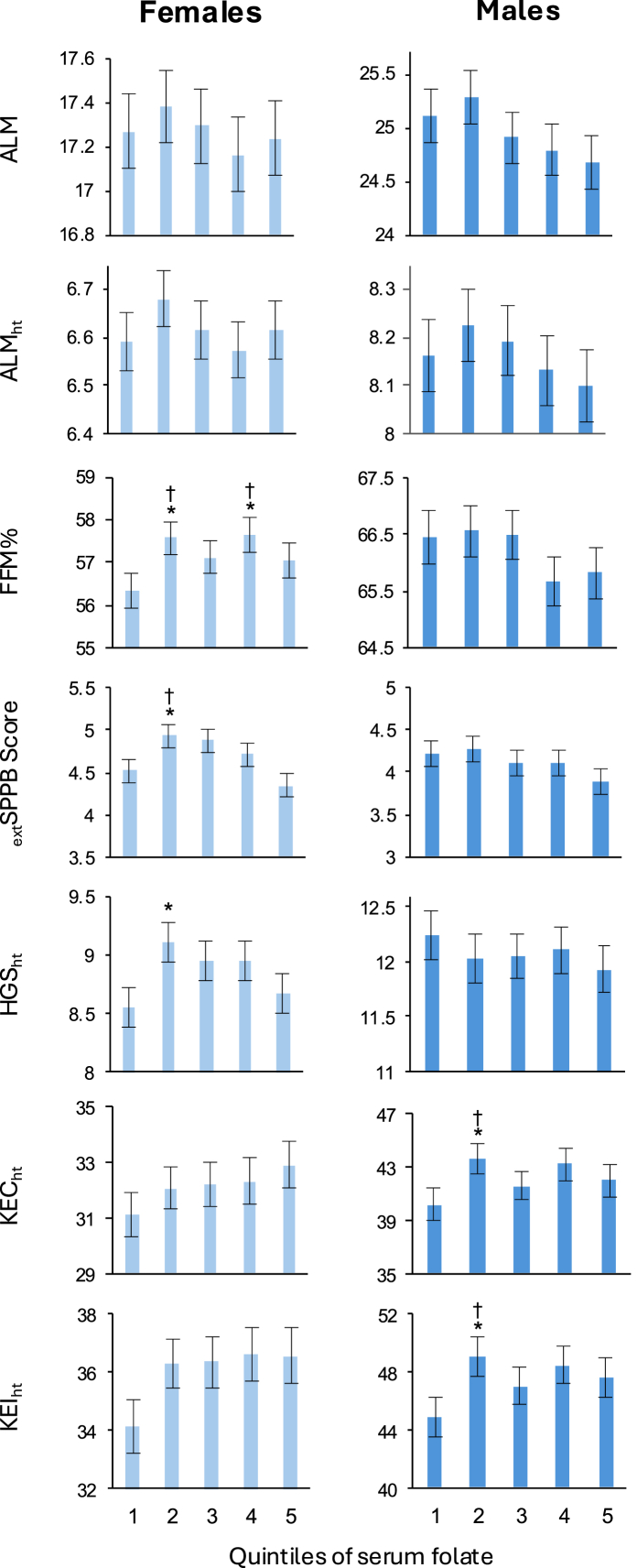
Adjusted means for muscle outcome measures by quintiles of serum folate concentration in females (left) and males (right). Data are presented as mean and SE (represented by vertical bars) calculated via linear regression. Extended short physical performance battery score (_ext_SPPB) was transformed using a Box-Cox transformation. All models were adjusted for age (years), smoking status (never, former, current), race (White American, Black American, and Other), physical activity level (not active, moderately active, active, and highly active), estimated glomerular filtration rate (mL/min/1.73 m^2^) and use of folate supplements (none, <400 μg/d, *≥*400 g/d, unknown). Appendicular lean mass (ALM) models were additionally adjusted for appendicular fat mass (kg) and height (m). ALM adjusted for height (ALM_ht_), fat-free mass percent (FFM%), _ext_SPPB, height-adjusted grip strength (HGS_ht_), and height-adjusted knee extension concentric (KEC_ht_) and isometric (KEI_ht_) strength models were additionally adjusted for BMI (kg/m^2^). ∗*P* < 0.05. † indicates that a significant association was also found when models included older adults only (≥65 y).

Full results of associations between nutritional biomarkers and lean mass outcomes can be found in Supplemental Tables 8 and 9, and forest plots of standardized regression coefficients (95% CI) for fully adjusted models in Figure 3. Results for folate are presented separately in Figure 4 (fully adjusted models) and Supplemental Tables 10 and 11. In fully adjusted (nonstandardized) models, vitamin B_12_ was positively associated with ALM_ht_ in females (*β* = 0.00017, *P* = 0.035). Negative associations were found between higher calcium and ALM (*β* = –0.37, *P* = 0.031; *β* = –0.60, *P* = 0.011) and ALM_ht_ (*β* = –0.16, *P* = 0.008; *β* = –0.20, *P* = 0.006) in females and males, respectively. In males, iron was positively associated with ALM_ht_ (*β* = 0.0021, *P* = 0.038). Compared with Q1 of serum folate, females in Q2 (*β* = 1.24, *P* = 0.025) and Q4 (*β* = 1.32, *P* = 0.019) had higher FFM%.

### Nutritional biomarkers and muscle strength and function

In fully adjusted (nonstandardized) models, in both males and females, higher magnesium was positively associated with _ext_SPPB (*β* = 0.85, *P* = 0.009 for both), and higher calcium was negatively associated with _ext_SPPB (*β* = –0.73, *P* < 0.001; *β* = –0.28, *P* = 0.048, respectively). Iron was positively associated with _ext_SPPB in males only (*β* = 0.0043, *P* = 0.045). Positive associations were found between potassium and KEC_ht_ in males (*β* = 4.85, *P* = 0.006) and with KEI_ht_ in males (*β* = 6.08, *P* = 0.002) and in females (*β* = 3.00, *P* = 0.041) (Supplemental Tables 12 and 13, Figure 3). Compared with Q1 of serum folate, females in Q2 had higher _ext_SPPB (*β* = 0.41, *P* = 0.038) and HGS_ht_ (*β* = 0.56, *P* = 0.018), and males in Q2 had higher KEC_ht_ (*β* = 3.42, *P* = 0.043) and KEI_ht_ (*β* = 4.16, *P* = 0.028) (Figure 4, Supplemental Tables 10 and 11).

### Biomarkers and muscle outcomes in older adults

For the significant associations described previously, FIGURE 2, FIGURE 3, FIGURE 4 show where these associations were also found in older adults only (≥65 y; marked with “†”). Most of the associations found between biomarkers and muscle outcomes in all males were also significant in males ≥65 y, with the exception of magnesium, iron and _ext_SPPB, and (in males with normal eGFR) creatinine and HGS_ht_. In older females, for lean mass measures, only the negative association between hemoglobin and ALM_ht_ was no longer significant. However, most associations between biomarkers and muscle strength or function were not found to be significant in older females (Supplemental Tables 14 and 15). For further information, see the Supplemental Results.

## Discussion

To our knowledge, this is the first study to investigate associations between a comprehensive range of clinical biochemistry and nutritional biomarkers and all 3 sarcopenic indices in males and females across a wide age range. We found that several clinical biochemistry biomarkers and measures of iron status were associated with sarcopenic indices. Fewer associations were found for nutritional biomarkers, and there were some unexpected inverse associations. Overall, where associations were found between biomarkers and muscle outcomes, the effect sizes were small (standardized *β* from 0.05 to 0.10). Effect sizes were slightly higher, but still small, in males for the associations between hemoglobin and HGS_ht_, and potassium and both knee extension strength measures (standardized *β* from 0.11 to 0.14). The largest standardized *β* coefficients were found between creatinine and muscle outcomes (in both males and females) when participants with low eGFR were excluded from the analyses (standardized *β* from 0.15 to 0.27). Therefore—with the exception of creatinine in participants with normal eGFR—the associations between biomarkers and muscle outcomes were generally small and similar in effect size in both males and females. However, these may be cumulatively important over time, or may be larger in older, less healthy populations.

### Measures of iron status

Hemoglobin is used in the diagnosis of anemia and the main cause of nutritional deficiency anemia is iron deficiency [37]. In males, hemoglobin was positively associated with ALM, HGS_ht_, and _ext_SPPB, whereas in females, hemoglobin was positively associated with _ext_SPPB, but negatively associated with ALM_ht_, although this negative association was not found in females aged ≥65 y. In the full cohort, more males (13.8% compared with 3.5%) had low hemoglobin, and more females (5.4% compared with 1.5%) had elevated hemoglobin levels, which may explain these gender differences. Additionally, in females, ALM_ht_ and _ext_SPPB were only very weakly correlated, which may partly explain these contrasting results. Previous studies have shown positive associations between hemoglobin and HGS [38,39] and physical function [[38], [39], [40]]. Serum iron reflects iron concentration in the blood, whereas serum ferritin is a measure of iron stores. Here, iron was positively associated with ALM_ht_ and _ext_SPPB in males, and ferritin was positively associated with KEC_ht_ and KEI_ht_ in females in the full cohort. In males and females aged ≥65 y, the associations with _ext_SPPB, KEC_ht_, and KEI_ht_ were not found to be significant. Two prior studies found no association between serum iron and HGS [41] or physical function [42]; however, both studies investigated associations in males and females together, and one study found a positive association between ferritin and HGS [41].

### Clinical biochemistry biomarkers

Albumin is the most abundant protein found in blood and is used in the evaluation of kidney and liver function. Prior studies have demonstrated positive, negative, and no significant associations between albumin and muscle mass [[43], [44], [45], [46], [47]]. Similarly, mixed results have been found for muscle strength and function [43,45,[47], [48], [49]]. Aging is associated with increased low-grade systemic inflammation, which may negatively impact both albumin levels and muscle mass [50,51]. It may be the case that these processes can co-occur but are not necessarily causally related. In the current study, we found negative associations between albumin and ALM, ALM_ht_, and _ext_SPPB in males and females, and a positive association with KEI_ht_ in females. In females aged ≥65 y, albumin was not associated with _ext_SPPB or KEI_ht_. The contrasting results in all females may be partly explained by the weak-to-moderate correlations found between these measures. These results are consistent with prior reports that show mixed relationships between albumin and measures related to sarcopenia.

Serum creatinine is also used to evaluate kidney function. Creatinine is influenced by muscle mass, and, although it has been suggested as a potential biomarker for sarcopenia [9], it has mainly been investigated in specific disease states [[52], [53], [54]]. In the current study, positive associations were found between creatinine and lean mass measures in males and females. Positive associations were also found between creatinine and muscle strength and function in males with normal eGFR. Further research in healthy populations may determine whether serum creatinine is useful as a risk or diagnostic biomarker for sarcopenia.

HbA1c is a diagnostic biomarker for diabetes and is used to evaluate glycemic control. We found that HbA1c was negatively associated with ALM_ht_, _ext_SPPB, and HGS_ht_ in males. Higher HbA1c was associated with sarcopenia [55] and lower HGS [48] in diabetic populations, but associations in nondiabetic populations appear relatively unexplored. Given the high prevalence rates of both diabetes (∼29%) and prediabetes (∼49%) in older adults [56], this may be cause for concern. Low HbA1c has been associated with micronutrient deficiency [57] and all-cause mortality in diabetic [58] and nondiabetic [59] populations, but a definitive cut-off point for “low” HbA1c has not been established. Further research is required to determine the optimal range of HbA1c in diabetic and nondiabetic populations, and whether measurements outside of this range are associated with sarcopenia.

### Nutritional biomarkers

Serum magnesium is tightly controlled, and individuals with deficiency or excess may present with normal serum concentrations [22]. Serum magnesium may be assessed in relation to a range of diseases, including kidney disease. In this study, magnesium was positively associated with _ext_SPPB in males and females, but no significant association was found in males aged ≥ 65 y. Previous studies investigating muscle strength have shown mixed results [22,60], perhaps because of this measure's limited capacity to capture nutritional status. Several studies have shown associations between dietary magnesium and sarcopenic indices [[61], [62], [63], [64]], and an intervention study found that daily magnesium supplementation, in addition to exercise, significantly improved measures of physical function, but not muscle strength [65].

Serum calcium is also assessed in relation to a range of clinical conditions including kidney function and bone health. Here, serum calcium was negatively associated with ALM, ALM_ht_, and _ext_SPPB in males and females, although an association with _ext_SPPB was not found in females aged ≥ 65 y. In contrast, 1 prior study found that higher albumin-adjusted serum calcium was associated with a lower risk of loss of muscle mass [66]. Although serum calcium is tightly controlled [67], the use of vitamin D and calcium supplements can lead to small but significant increases [68]. In this cohort, a large proportion of participants, particularly older participants, supplemented with vitamin D or calcium, potentially increasing calcium concentrations in those more likely to have lower muscle mass, strength, or function.

Serum vitamin D is a measure of nutritional status, and low vitamin D levels have been associated with larger decreases in muscle strength [69,70] and physical function [70]. An umbrella review of intervention trials of vitamin D to improve sarcopenic indices recommended supplementation only for individuals with vitamin D deficiency [71]. In this cohort, a smaller number of participants were deficient (0.8%) or moderately deficient (9.5%) in vitamin D as compared with the wider United States population (2.6% and 22.0%, respectively) [72], which may explain why no significant associations were found.

We found a positive association between serum B_12_ and ALM_ht_ in females. Serum vitamin B_12_ is used as a measure of nutritional status. Both low serum B_12_ and low dietary intakes of B_12_ have been associated with sarcopenia [[73], [74], [75], [76]], but studies investigating serum B_12_ and sarcopenic indices have shown both positive [73,77,78] and no associations [[79], [80], [81]]. Potential differences in the proportion of participants with low or deficient B_12_ status in a given population may have contributed to these mixed results.

Serum potassium was positively associated with KEC_ht_ in males, and KEI_ht_ in males and females (but not females aged ≥65 y). To our knowledge, no previous studies have investigated associations between serum potassium and sarcopenic indices. Similar to calcium and magnesium, serum potassium concentration is tightly controlled and is unlikely to reflect nutritional status [82], but is often assessed routinely in healthcare and in relation to kidney function or hypertension. Some previous studies have shown associations between dietary potassium and sarcopenia [75] or sarcopenic indices [[83], [84], [85]], but others have not [64,76].

Serum folate is used as a measure of nutritional status. In this study, Q2 compared with Q1 of serum folate concentration was associated with higher FFM%, extSPPB and in females, and with higher knee extension strength in males, in the full cohort and in those aged ≥65 y. Higher concentrations of serum folate (Q3–Q5) were generally not associated with better muscle outcomes. United States cohorts are likely to have higher serum folate than non-United States cohorts because of United States food fortification practices [86]. Additionally, over half of our participants used folate supplements and none had below-normal serum folate. Three previous studies found positive associations between serum folate and muscle strength. Two reported linear associations [79,87], and the third compared participants with folate concentrations above the population mean to those with folate concentrations below the population mean. [88]. Mean folate concentrations were far lower in these 3 studies (<9.6 ng/mL), conducted in Asian populations [79,87,88], compared with the median (19.5 ng/mL) and mean (23.2 ng/mL) concentrations found in our participants. A fourth study in United States participants (mean serum folate 19.4 ng/mL) investigated quartiles of serum folate, finding that BMI-adjusted grip strength was higher in quartile 3 compared with quartile 1 only [89]. These results suggest that maintaining adequate folate concentrations may be important for muscle health, but that further increasing concentrations may not be beneficial.

### Strengths and limitations

This study’s strengths include the use of a large cohort of healthy adults spanning a wide age range, including the full spectrum of age over which age-related declines in muscle health have been observed. This allows for associations between biomarkers and muscle outcomes to be investigated in a population largely free of sarcopenia, highlighting biomarkers that are associated with declining muscle health (and potentially increased risk of sarcopenia) before sarcopenia develops. Both nonstandardized and standardized results were provided for biomarkers treated as continuous variables to allow for the comparison of results between different biomarkers. Analyses were stratified by gender to account for gender differences in the effect of aging on skeletal muscle, and we included the full range of sarcopenic indices as outcome measures. We investigated biomarkers that are readily available for use in future research or in clinical practice, and use of nutritional biomarkers avoids potential reporting errors that may be present with dietary consumption data (for nutritional biomarkers that are reflective of nutritional status). Associations between micronutrients and muscle outcomes observed in other studies may not be present in this cohort because of the generally healthy status and the proportion and characteristics of participants using dietary supplements. Additionally, supplement use information was lacking for over a third of participants, limiting our ability to accurately adjust for this potential confounder. Finally, because of the large number of models included in our analyses, there may be an increased risk of type 1 errors (false positives) that have occurred by chance. The use of methods to adjust for multiple testing in epidemiological and exploratory studies is debated, and there is a lack of consensus on exactly when or how this should be done [[90], [91], [92]]. However, our intention was to understand the relationships between a wider range of individual measures of clinical biochemistry and nutritional biomarkers, with sarcopenic indices, than had previously been researched, and so we feel that this also justifies our approach. Ultimately, further research will be required to support our findings.

In conclusion, this study contributes to a growing body of evidence that routinely measured biomarkers may be useful for identifying individuals at risk of sarcopenia. Serum creatinine and measures of iron status appear to be of importance, particularly in males. Longitudinal studies will provide additional evidence in this area, and intervention studies that address the underlying cause of alterations in clinical biomarker measures, or improve nutritional status in individuals with micronutrient deficiencies, are needed to establish a causal connection between these and sarcopenia. This could lead to the development of nutritional or other therapeutic strategies that could be used to prevent, manage, or treat sarcopenia.

## Author contributions

The authors’ responsibilities were as follows – JS, DC, MY, LF, AAW: designed research; TT: provided essential materials; JS: performed statistical analysis; JS, DC, MY, AAW: wrote the paper; JS, AAW: primary responsibility for the final content; and all authors: read, contributed, and approved the final manuscript.

## Data availability

The raw data supporting the conclusion of this manuscript will be made available without undue reservation, by the authors, to any qualified researcher, provided that they submit a research proposal on the Baltimore Longitudinal Study of Aging website: https://www.blsa.nih.gov/how-apply.

## Funding

This work was supported by the Wellcome Trust EDESIA PhD Programme (218467/Z/19/Z). The funders had no role in the study design, data collection, data analysis, decision to publish or preparation of the manuscript.

## Conflict of interest

The authors report no conflicts of interest.

## References

[1] Wolfe R.R. (2006). The underappreciated role of muscle in health and disease. Am. J. Clin. Nutr..

[2] Argilés J.M., Campos N., Lopez-Pedrosa J.M., Rueda R., Rodriguez-Mañas L. (2016). Skeletal muscle regulates metabolism via interorgan crosstalk: roles in health and disease. J. Am. Med. Dir. Assoc..

[3] Merz K.E., Thurmond D.C. (2020). Role of skeletal muscle in insulin resistance and glucose uptake, Compr. Physiol.

[4] Petermann-Rocha F., Balntzi V., Gray S.R., Lara J., Ho F.K., Pell J.P. (2022). Global prevalence of sarcopenia and severe sarcopenia: a systematic review and meta-analysis. J. Cachexia Sarcopenia Muscle.

[5] Goodpaster B.H., Park S.W., Harris T.B., Kritchevsky S.B., Nevitt M., Schwartz A.V. (2006). The loss of skeletal muscle strength, mass, and quality in older adults: the health, aging and body composition study. J. Gerontol. A. Biol. Sci. Med. Sci..

[6] Du Y., Wang X., Xie H., Zheng S., Wu X., Zhu X. (2019). Sex differences in the prevalence and adverse outcomes of sarcopenia and sarcopenic obesity in community dwelling elderly in East China using the AWGS criteria. BMC Endocr. Disord..

[7] Welch A.A., Hayhoe R.P.G., Cameron D. (2020). The relationships between sarcopenic skeletal muscle loss during ageing and macronutrient metabolism, obesity and onset of diabetes. Proc. Nutr. Soc..

[8] Ethgen O., Beaudart C., Buckinx F., Bruyère O., Reginster J.Y. (2017). The future prevalence of sarcopenia in Europe: a claim for public health action. Calcif. Tissue Int..

[9] Curcio F., Ferro G., Basile C., Liguori I., Parrella P., Pirozzi F. (2016). Biomarkers in sarcopenia: a multifactorial approach. Exp. Gerontol..

[10] Calvani R., Marini F., Cesari M., Tosato M., Picca A., Anker S.D. (2017). Biomarkers for physical frailty and sarcopenia. Aging Clin. Exp. Res..

[11] Lian R., Liu Q., Jiang G., Zhang X., Tang H., Lu J. (2024). Blood biomarkers for sarcopenia: a systematic review and meta-analysis of diagnostic test accuracy studies. Ageing Res. Rev..

[12] Picca A., Coelho-Junior H.J., Calvani R., Marzetti E., Vetrano D.L. (2022). Biomarkers shared by frailty and sarcopenia in older adults: a systematic review and meta-analysis. Ageing Res. Rev..

[13] Landi F., Camprubi-Robles M., Bear D.E., Cederholm T., Malafarina V., Welch A.A. (2019). Muscle loss: the new malnutrition challenge in clinical practice. Clin. Nutr..

[14] Welch A., Hayhoe R., Veronese N., Beaudart C., Sabico S. (2021). Sarcopenia: Research and Clinical Implications.

[15] Smith G.I., Mittendorfer B. (2016). Sexual dimorphism in skeletal muscle protein turnover. J. Appl. Physiol..

[16] Shock N.W., Greulich R.C., Aremberg D., Costa P.T., Lakatta E.G., Tobin J.D. (1984).

[17] Cruz-Jentoft A.J., Bahat G., Bauer J., Boirie Y., Bruyère O., Cederholm T. (2019). Sarcopenia: revised European consensus on definition and diagnosis. Age Ageing.

[18] Studenski S.A., Peters K.W., Alley D.E., Cawthon P.M., McLean R.R., Harris T.B. (2014). The FNIH sarcopenia project: rationale, study description, conference recommendations, and final estimates. J. Gerontol. A Biol. Sci. Med. Sci..

[19] Fielding R.A., Vellas B., Evans W.J., Bhasin S., Morley J.E., Newman A.B. (2011). Sarcopenia: an undiagnosed condition in older adults. Current consensus definition: prevalence, etiology, and consequences. International working group on sarcopenia, J. Am. Med. Dir. Assoc..

[20] Chen L.K., Woo J., Assantachai P., Auyeung T.W., Chou M.Y., Iijima K. (2020). Asian Working Group for Sarcopenia: 2019 Consensus Update on Sarcopenia Diagnosis and Treatment. J. Am. Med. Dir. Assoc..

[21] Osawa Y., Tian Q., An Y., Studenski S.A., Resnick S.M., Ferrucci L. (2021). Longitudinal associations between brain volume and knee extension peak torque. J. Gerontol. A Biol. Sci. Med. Sci..

[22] Cameron D., Welch A.A., Adelnia F., Bergeron C.M., Reiter D.A., Dominguez L.J. (2019). Age and muscle function are more closely associated with intracellular magnesium, as assessed by (31)P magnetic resonance spectroscopy, than with serum magnesium. Front. Physiol..

[23] Simonsick E.M., Newman A.B., Nevitt M.C., Kritchevsky S.B., Ferrucci L., Guralnik J.M. (2001). Measuring higher level physical function in well-functioning older adults: expanding familiar approaches in the Health ABC Study. J. Gerontol. A Biol. Sci. Med. Sci..

[24] Ubaida-Mohien C., Gonzalez-Freire M., Lyashkov A., Moaddel R., Chia C.W., Simonsick E.M. (2019). Physical activity associated proteomics of skeletal muscle: being physically active in daily life may protect skeletal muscle from aging. Front. Physiol..

[25] Degens H., Gayan-Ramirez G., van Hees H.W. (2015). Smoking-induced skeletal muscle dysfunction: from evidence to mechanisms. Am. J. Respir. Crit. Care Med..

[26] Cheng T.C., Huang S.H., Kao C.L., Hsu P.C. (2022). Muscle wasting in chronic kidney disease: mechanism and clinical implications—a narrative review. Int. J. Mol. Sci..

[27] Inker L.A., Eneanya N.D., Coresh J., Tighiouart H., Wang D., Sang Y. (2021). New creatinine- and cystatin C-based equations to estimate GFR without race. N. Engl. J. Med..

[28] John W.G. (2012). UK Department of Health Advisory Committee on Diabetes, Use of HbA1c in the diagnosis of diabetes mellitus in the UK. The implementation of World Health Organization guidance 2011, Diabet. Med.

[29] Portolès J., Martín L., Broseta J.J., Cases A. (2021). Anemia in chronic kidney disease: from pathophysiology and current treatments, to future agents. Front. Med. (Lausanne)..

[30] Hassanein M., Shafi T. (2022). Assessment of glycemia in chronic kidney disease. BMC Med.

[31] Kim C.S., Kim S.W. (2014). Vitamin D and chronic kidney disease. Korean J. Intern. Med..

[32] McMahon G.M., Hwang S., Tanner R.M., Jacques P.F., Selhub J., Muntner P. (2015). The association between vitamin B12, albuminuria and reduced kidney function: an observational cohort study. BMC Nephrol.

[33] Hill Gallant K.M., Spiegel D.M. (2017). Calcium balance in chronic kidney disease. Curr. Osteoporos. Rep..

[34] Dhondup T., Qian Q. (2017). Acid-base and electrolyte disorders in patients with and without chronic kidney disease: an update. Kidney Dis. (Basel).

[35] Tomasz G., Ewa W., Jolanta M. (2021). Biomarkers of iron metabolism in chronic kidney disease. Int. Urol. Nephrol..

[36] Wang A., Yeung L.F., Ríos Burrows N., Rose C.E., Fazili Z., Pfeiffer C.M. (2022). Reduced kidney function is associated with increasing red blood cell folate concentration and changes in folate form distributions (NHANES 2011–2018). Nutrients.

[37] Beard J.L. (2001). Iron biology in immune function, muscle metabolism and neuronal functioning. J. Nutr..

[38] Hirani V., Naganathan V., Blyth F., Le Couteur D.G., Seibel M.J., Waite L.M. (2016). Low hemoglobin concentrations are associated with sarcopenia, physical performance, and disability in older Australian men in cross-sectional and longitudinal analysis: the Concord Health and Ageing in Men Project. J. Gerontol. A Biol. Sci. Med. Sci..

[39] Cecchi F., Pancani S., Vannetti F., Boni R., Castagnoli C., Paperini A. (2017). Hemoglobin concentration is associated with self-reported disability and reduced physical performance in a community dwelling population of nonagenarians: the Mugello Study, Intern. Emerg. Med..

[40] Chaves P.H.M., Ashar B., Guralnik J.M., Fried L.P. (2002). Looking at the relationship between hemoglobin concentration and prevalent mobility difficulty in older women. Should the criteria currently used to define anemia in older people be reevaluated?. J. Am. Geriatr. Soc..

[41] Ho V., Lee C.T., Merchant R.A. (2022). The “Iron Tale” - iron indices and handgrip strength in community-dwelling adults. Aging Clin. Exp. Res..

[42] Bartali B., Frongillo E.A., Guralnik J.M., Stipanuk M.H., Allore H.G., Cherubini A. (2008). Serum micronutrient concentrations and decline in physical function among older persons. JAMA.

[43] Reijnierse E.M., Trappenburg M.C., Leter M.J., Sipilä S., Stenroth L., Narici M.V. (2015). Serum albumin and muscle measures in a cohort of healthy young and old participants. Age (Dordr).

[44] Chen Z., Song C., Yao Z., Sun J., Liu W. (2022). Associations between albumin, globulin, albumin to globulin ratio and muscle mass in adults: results from the national health and nutrition examination survey 2011–2014. BMC Geriatr.

[45] van Atteveld V.A., Van Ancum J.M., Reijnierse E.M., Trappenburg M.C., Meskers C.G.M., Maier A.B. (2019). Erythrocyte sedimentation rate and albumin as markers of inflammation are associated with measures of sarcopenia: a cross-sectional study. BMC Geriatr.

[46] Visser M., Kritchevsky S.B., Newman A.B., Goodpaster B.H., Tylavsky F.A., Nevitt M.C. (2005). Lower serum albumin concentration and change in muscle mass: the Health, Aging and Body Composition Study. Am. J. Clin. Nutr..

[47] Snyder C.K., Lapidus J.A., Cawthon P.M., Dam T.T., Sakai L.Y., Marshall L.M. (2012). Serum albumin in relation to change in muscle mass, muscle strength, and muscle power in older men. J. Am. Geriatr. Soc..

[48] Yokoyama H., Shiraiwa T., Takahara M., Iwamoto M., Kuribayashi N., Nomura T. (2020). Applications of physical performance measures to routine diabetes care for frailty prevention concept: fundamental data with grip strength, gait speed, timed chair stand speed, standing balance, and knee extension strength. BMJ Open Diabetes Res. Care.

[49] Schalk B.W.H., Deeg D.J.H., Penninx B.W.J.H., Bouter L.M., Visser M. (2005). Serum albumin and muscle strength: a longitudinal study in older men and women. J. Am. Geriatr. Soc..

[50] Wiedermann C.J. (2021). Hypoalbuminemia as surrogate and culprit of infections. Int. J. Mol. Sci..

[51] Dalle S., Rossmeislova L., Koppo K. (2017). The role of inflammation in age-related sarcopenia. Front. Physiol..

[52] Patel S.S., Molnar M.Z., Tayek J.A., Ix J.H., Noori N., Benner D. (2013). Serum creatinine as a marker of muscle mass in chronic kidney disease: results of a cross-sectional study and review of literature. J. Cachexia Sarcopenia Muscle.

[53] Holdom C.J., Janse van Mantgem M.R., van Eijk R.P.A., Howe S.L., van den Berg L.H., McCombe P.A. (2021). Venous creatinine as a biomarker for loss of fat-free mass and disease progression in patients with amyotrophic lateral sclerosis. Eur. J. Neurol..

[54] Zhi J., Jiāo B., Qing S., Liang L. (2023). Factors associated with low skeletal muscle index among patients with Crohn's disease. Rev. Assoc. Med. Bras. (1992).

[55] Lin Y., Zhang Y., Shen X., Huang L., Yan S. (2021). Influence of glucose, insulin fluctuation, and glycosylated hemoglobin on the outcome of sarcopenia in patients with type 2 diabetes mellitus. J. Diabetes Complications.

[56] Centers for Disease Control and Prevention (2023). National Diabetes Statistics Report [Internet]. https://www.cdc.gov/diabetes/php/data-research/?CDC_AAref_Val=https://www.cdc.gov/diabetes/data/statistics-report/index.html.

[57] Truijen S.P.M., Hayhoe R.P.G., Hooper L., Schoenmakers I., Forbes A., Welch A.A. (2021). Predicting malnutrition risk with data from routinely measured clinical biochemical diagnostic tests in free-living older populations. Nutrients.

[58] Abdelhafiz A.H., Sinclair A.J. (2015). Low HbA1c and increased mortality risk-is frailty a confounding factor?. Aging Dis.

[59] Carson A.P., Fox C.S., McGuire D.K., Levitan E.B., Laclaustra M., Mann D.M. (2010). Low hemoglobin A1c and risk of all-cause mortality among US adults without diabetes. Circ. Cardiovasc. Qual. Outcomes..

[60] Dominguez L.J., Barbagallo M., Lauretani F., Bandinelli S., Bos A., Corsi A.M. (2006). Magnesium and muscle performance in older persons: the InCHIANTI study. Am. J. Clin. Nutr..

[61] Hayhoe R.P.G., Lentjes M.A.H., Mulligan A.A., Luben R.N., Khaw K.T., Welch A.A. (2019). Cross-sectional associations of dietary and circulating magnesium with skeletal muscle mass in the EPIC-Norfolk cohort. Clin. Nutr..

[62] Welch A.A., Kelaiditi E., Jennings A., Steves C.J., Spector T.D., MacGregor A. (2016). Dietary magnesium is positively associated with skeletal muscle power and indices of muscle mass and may attenuate the association between circulating C-reactive protein and muscle mass in women. J. Bone Miner. Res..

[63] Welch A.A., Skinner J., Hickson M. (2017). Dietary magnesium may be protective for aging of bone and skeletal muscle in middle and younger older age men and women: cross-sectional findings from the UK biobank cohort. Nutrients.

[64] Gedmantaite A., Celis-Morales C.A., Ho F., Pell J.P., Ratkevicius A., Gray S.R. (2020). Associations between diet and handgrip strength: a cross-sectional study from UK Biobank. Mech. Ageing Dev..

[65] Veronese N., Berton L., Carraro S., Bolzetta F., De Rui M., Perissinotto E. (2014). Effect of oral magnesium supplementation on physical performance in healthy elderly women involved in a weekly exercise program: a randomized controlled trial. Am. J. Clin. Nutr..

[66] Kim Y.S., Hong K.W., Han K., Park Y.C., Park J.M., Kim K. (2020). Longitudinal observation of muscle mass over 10 years according to serum calcium levels and calcium intake among korean adults aged 50 and older: the Korean Genome and Epidemiology Study. Nutrients.

[67] Zheng J., Zeng X., Wang S. (2015). Calcium ion as cellular messenger. Sci. China Life Sci..

[68] Yuan S., Baron J.A., Michaëlsson K., Larsson S.C. (2021). Serum calcium and 25-hydroxyvitamin D in relation to longevity, cardiovascular disease and cancer: a Mendelian randomization study. NPJ Genom. Med.

[69] Mizuno T., Hosoyama T., Tomida M., Yamamoto Y., Nakamichi Y., Kato S. (2022). Influence of vitamin D on sarcopenia pathophysiology: a longitudinal study in humans and basic research in knockout mice. J. Cachexia Sarcopenia Muscle.

[70] Conzade R., Grill E., Bischoff-Ferrari H.A., Ferrari U., Horsch A., Koenig W. (2019). Vitamin D in relation to incident sarcopenia and changes in muscle parameters among older adults: the KORA-Age Study, Calcif. Tissue Int.

[71] De Spiegeleer A., Beckwée D., Bautmans I., Petrovic M. (2018). Sarcopenia guidelines development group of the Belgian Society of Gerontology and Geriatrics (BSGG), pharmacological interventions to improve muscle mass, muscle strength and physical performance in older people: an umbrella review of systematic reviews and meta-analyses. Drugs Aging.

[72] Cui A., Xiao P., Ma Y., Fan Z., Zhou F., Zheng J. (2022). Prevalence, trend, and predictor analyses of vitamin D deficiency in the US population, 2001–2018. Front. Nutr..

[73] Ates Bulut E., Soysal P., Aydin A.E., Dokuzlar O., Kocyigit S.E., Isik A.T. (2017). Vitamin B12 deficiency might be related to sarcopenia in older adults. Exp. Gerontol..

[74] Choi S., Chon J., Lee S.A., Yoo M.C., Chung S.J., Shim G.Y. (2023). Impact of vitamin B12 insufficiency on the incidence of sarcopenia in Korean community-dwelling older adults: a Two-year longitudinal study. Nutrients.

[75] Petermann-Rocha F., Chen M., Gray S.R., Ho F.K., Pell J.P., Celis-Morales C. (2020). Factors associated with sarcopenia: a cross-sectional analysis using UK Biobank. Maturitas.

[76] Santiago E.C.S., Roriz A.K.C., Ramos L.B., Ferreira A.J.F., Oliveira C.C., Gomes-Neto M. (2021). Comparison of calorie and nutrient intake among elderly with and without sarcopenia: a systematic review and meta-analysis. Nutr. Rev..

[77] Chae S.A., Kim H.S., Lee J.H., Yun D.H., Chon J., Yoo M.C. (2021). Impact of vitamin B12 insufficiency on sarcopenia in community-dwelling older Korean adults. Int. J. Environ. Res. Public Health..

[78] Soh Y., Won C.W. (2020). Association between frailty and vitamin B12 in the older Korean population. Medicine (Baltimore).

[79] Ao M., Inuiya N., Ohta J., Kurose S., Takaoka H., Abe Y. (2019). Relationship between homocysteine, folate, vitamin B12 and physical performance in the institutionalized elderly. J. Nutr. Sci. Vitaminol. (Tokyo).

[80] Ng T.P., Aung K.C.Y., Feng L., Scherer S.C., Yap K.B. (2012). Homocysteine, folate, vitamin B-12, and physical function in older adults: cross-sectional findings from the Singapore Longitudinal Ageing Study. Am. J. Clin. Nutr..

[81] Vidoni M.L., Pettee Gabriel K., Luo S.T., Simonsick E.M., Day R.S. (2017). Vitamin B12 and homocysteine associations with gait speed in older adults: the Baltimore Longitudinal Study of Aging. J. Nutr. Health Aging.

[82] Gumz M.L., Rabinowitz L., Wingo C.S. (2015). An integrated view of potassium homeostasis. N. Engl. J. Med..

[83] Lee Y.J., Lee M., Wi Y.M., Cho S., Kim S.R. (2020). Potassium intake, skeletal muscle mass, and effect modification by sex: data from the 2008–2011 KNHANES. Nutr. J..

[84] Tak Y.J., Lee J.G., Yi Y.H., Kim Y.J., Lee S., Cho B.M. (2018). Association of handgrip strength with dietary intake in the Korean Population: findings based on the Seventh Korea National Health and Nutrition Examination Survey (KNHANES VII-1), 2016. Nutrients.

[85] Kim N.H., Kim C.Y. (2022). Association of micronutrients and handgrip strength in Korean older population: a cross-sectional study. Healthcare (Basel).

[86] Tanaka T., Scheet P., Giusti B., Bandinelli S., Piras M.G., Usala G. (2009). Genome-wide association study of vitamin B6, vitamin B12, folate, and homocysteine blood concentrations. Am. J. Hum. Genet..

[87] Wee A.K.H. (2016). Serum folate predicts muscle strength: a pilot cross-sectional study of the association between serum vitamin levels and muscle strength and gait measures in patients >65 years old with diabetes mellitus in a primary care setting. Nutr. J..

[88] Lee M.R., Jung S.M. (2021). Folic acid is related to muscle strength and vitamin A is related to health-related quality of life: results of the Korea National Health and Nutrition Examination Survey (KNHANES VII 2016–2018). Nutrients.

[89] Zhang L., Sun J., Li Z., Zhang D. (2021). The relationship between serum folate and grip strength in American adults. Arch. Osteoporos..

[90] Bender R., Lange S. (2001). Adjusting for multiple testing—when and how?. J. Clin. Epidemiol..

[91] Perneger T.V. (1998). What's wrong with Bonferroni adjustments. BMJ.

[92] Rothman K.J. (1990). No adjustments are needed for multiple comparisons. Epidemiology.

