# Supplementary material for: Cross-Sectional Associations between Clinical Biochemistry and Nutritional Biomarkers and Sarcopenic Indices of Skeletal Muscle in the Baltimore Longitudinal Study of Aging
Source: J Nutr. 2025 Mar 8;155(5):1535–48. doi: 10.1016/j.tjnut.2025.03.006 (PMC12121409; doi:10.1016/j.tjnut.2025.03.006)
Supplement: Multimedia component 1 [file mmc1.docx]

**Title:** Cross-sectional associations between clinical biochemistry and nutritional biomarkers and sarcopenic indices of skeletal muscle in the Baltimore Longitudinal Study of Aging.

**Author Names:** Jamie Scott, Max Yates, Toshiko Tanaka, Luigi Ferrucci, Donnie Cameron and Ailsa A. Welch

**Corresponding Author:** Jamie Scott, Norwich Medical School, University of East Anglia, Norwich, UK, NR4 7TJ. Email address: [J.Scott2@uea.ac.uk](mailto:J.Scott2@uea.ac.uk)

**Table of Contents**

| Supplementary Methods | Page 3 |
| --- | --- |
| Supplementary Results | Page 5 |
| References | Page 8 |
| Supplementary Table 1 | Page 10 |
| Supplementary Table 2 | Page 11 |
| Supplementary Table 3 | Page 12 |
| Supplementary Table 4 | Page 13 |
| Supplementary Table 5 | Page 14 |
| Supplementary Table 6 | Page 15 |
| Supplementary Table 7 | Page 16 |
| Supplementary Table 8 | Page 17 |
| Supplementary Table 9 | Page 19 |
| Supplementary Table 10 | Page 21 |
| Supplementary Table 11 | Page 23 |
| Supplementary Table 12 | Page 25 |
| Supplementary Table 13 | Page 27 |
| Supplementary Table 14 | Page 29 |
| Supplementary Table 15 | Page 31 |
| Supplementary Figure 1 | Page 33 |
| Supplementary Figure 2 | Page 34 |

**Supplementary Methods**

***Dietary Supplement Use***

With the exception of potassium, dietary supplement use was categorised as ‘no supplement use’, low supplement use’, ‘high supplement use’, or ‘missing supplement use information’. For vitamin B12, folate (as dietary folate equivalents) and calcium, cut-off points for low/high supplement use were set at the US recommended daily allowance (RDA) for each nutrient for adults (2.4$\mu$g/day, 400$\mu$g/day, and 1,000mg/day respectively) [1]. The RDA for vitamin D in adults is 15 mg/day [1]: within this cohort, histograms of supplemental intake showed a gap between participants consuming less than 12mg/day, and around or above the RDA (lowest intake 14.7 mg/day). Therefore, a cut-off of 14.5mg/day for low/high supplement use was selected. The RDA for iron varies with age and gender, set at 18mg/day for women under 50 years old, and 8 mg/day for all men, and for women over 50 years old [1]. Within this cohort, all women under the age of 50 using iron supplements had supplemental intakes < 8 mg/day or > 18mg/day. A cut-off of 8 mg/day was chosen for low/high supplement use, as this assigned all participants with supplemental intakes above the RDA for their age and gender to the ‘high supplement use’ group. The RDA for magnesium in adults is 320 mg/day for women and 420 mg/day for men [1]. Histograms of supplemental magnesium showed that participants either consumed less than 100 mg/day, or $\geq$ 400 mg/day, therefore, a cut-off point of 400 mg/day was chosen for low/high supplement use. Potassium intake from supplements was below 200 mg/day in all participants, lower than the RDA of 2,600 mg/day in women and 3,400 mg/day in men [1]. Potassium supplementation was categorised as ‘no supplementation’, ‘supplement use’, or ‘missing supplement information’.

***Statistical Analysis***

All multiple regression models, except those containing creatinine, were adjusted for eGFR as poor renal function is associated with loss of muscle mass, strength and physical function [2]. Declining renal function can also affect the concentrations of many clinical and nutritional biomarkers in the body [3-10]. Because creatinine is used in the calculation of eGFR, there is a linear relationship between these variables and collinearity when these terms are simultaneously entered into regression models, therefore, a sensitivity analysis was completed to investigate associations between serum creatinine and muscle outcomes in individuals with normal eGFR (> 60 mL/min/1.73m^2^). All multiple linear regression models containing ferritin were adjusted for CRP, as serum ferritin is affected by inflammation. A cut-off of >5$\mu$g/mL CRP has been suggested as the level above which ferritin measures may be altered [11]. CRP was entered into the models as a binary variable ($\leq$ 5$\mu$g/mL and > 5$\mu$g/mL). Multivariable models containing ALM were adjusted for AFM and height as muscle mass increases with increased fat mass [12, 13] and height [14]. Multivariable models containing ALM_ht_ were adjusted for BMI as this measure accounts for height, but not body composition. Multivariable models containing FFM% were also adjusted for BMI as, although muscle mass increases with increased adiposity, the proportion of muscle mass decreases [13]. Here, BMI was standardised and entered as a quadratic term, to avoid collinearity between BMI and BMI^2^ terms, and to account for the non-linear relationship between FFM% and BMI. To meet linear regression assumptions, _ext_SPPB was normalised using a Box-Cox transformation: this requires that the variable contains no ‘0’ values. A small number of participants (13 men and 7 women) had _ext_SPPB scores of 0. In order to include all participants in the analysis, a small constant (0.1) was added to _ext_SPPB score for all participants before transformation. Multivariable models containing Box-Cox transformed _ext_SPPB were adjusted for age, which was standardised and entered as a quadratic term, to avoid collinearity between age and age^2^, and account for the non-linear relationship between _ext_SPPB and age. _ext_SPPB, HGS_ht_ and KE strength (KEC_ht_ and KEI_ht_) multivariable models were adjusted for BMI as adiposity can affect muscle strength [15] and function [16]. Nested model 3 for nutritional biomarkers were adjusted for dietary supplement use of the corresponding nutrient, as there may be underlying differences in the demographic characteristics of individuals who do or do not consume dietary supplements.

**Supplementary Results**

***Invalid/Missing Measurements***

Serum vitamin B12 measurements were missing for 150 participants . A further 35 serum B12 measurements, above the analytical range of detection of 50–2,000 pg/mL, were excluded from the dataset, resulting in 185 missing or invalid B12 measurements. Three participants had HGS measurements in one hand above the dynamometer’s measurement range of 0–90kg. In this instance, the valid measurement for the opposite hand was used. HGS, KEC and KEI measurements were missing for 15, 255 and 269 participants, respectively, on one side of the body. For these participants, the maximum measurement from the other side was used.

***Correlation Between Lean Mass, Muscle Strength and Muscle Function***

ALM and ALM_ht_ were strongly correlated in men (*r_s_*=0.85, *p* < 0.001) and women (*r_s_=*0.87, *p*<0.001), however, FFM% was very weakly positively and negatively correlated with ALM measures in men (ALM: *r_s_*=0.14, ALM_ht_: *r_s_*=0.18; *p* for both <0.001) and women (ALM: *r_s_*=-0.09, ALM_ht_: *r_s_*=-0.09; *p* for both <0.05) respectively. Of the lean mass measures, ALM was most strongly correlated with _ext_SPPB in men (*r_s_*=0.48, *p*<0.001), whereas FFM% was most strongly correlated with _ext_SPPB in women (*r_s_*=0.31, *p*<0.001). ALM was generally more strongly correlated with measures of muscle strength than other lean mass measures in both men (HGS_ht_: *r_s_*=0.55; KEC_ht:_ *r_s_*=0.58; KEI_ht_: *r_s_*=0.59; *p*<0.001 for all) and women (HGS_ht_: *r_s_*=0.39; KEC_ht:_ *r_s_*=0.36; KEI_ht_: *r_s_*=0.38; *p*<0.001 for all). For muscle strength and function measures, _ext_SPPB was more strongly correlated with HGS_ht_ in men (*r_s_*=0.60, *p*<0.001) than women (*r_s_*=0.53, p<0.001), but more strongly correlated with KEC_ht_ (*r_s_*=0.57 vs *r_s_*=0.48, *p* for both <0.001) and KEI_ht_ (*r_s_*=0.53 vs *r_s_*=0.45, *p* for both <0.001) in women than men, respectively. In both men and women, correlations between HGS_ht_ and KEC_ht_/KEI_ht_ were strong and similar (*r_s_* from 0.63-0.65, *p* for all <0.001), and correlations between KEC_ht_ and KEI_ht_ were very strong and similar (*r_s_* from 0.90-0.92, *p* for all <0.001).

***Biomarkers and Muscle Outcomes in Older Adults***

Where significant associations were found between clinical or nutritional biomarkers and muscle outcomes, we investigated whether these associations were present in participants aged ≥65 years (**Supplementary Tables 14 and 15**). All associations found between clinical biomarkers and lean mass remained significant in men ≥65 years. The negative association between haemoglobin and ALM_ht_ was no longer significant in women ≥65 years ($\beta$=-0.057, *p*=0.065). For clinical biomarkers and muscle strength and function measures, only the association between haemoglobin and _ext_SPPB remained significant in women ($\beta$=0.18, *p*=0.014). In men, only the association between creatinine (in participants with eGFR ≥60mL/min/1.73m^2^) and HGS_ht_ was no longer significant ($\beta$=1.50, *p*=0.10).

Where associations were found between nutritional biomarkers and measures of lean mass in all participants, these associations were also significant in participants ≥ 65 years old (*p* for all < 0.05). However, several of the associations found between nutritional biomarkers and muscle strength and function in the full cohort were not present in older adults. Although magnesium was associated with _ext_SPPB in both women and men in the full cohort, the association remained significant in women ($\beta$=0.97, *p*=0.013), but not in men ($\beta$=0.68, *p*=0.08) ≥ 65 years. The association between calcium and _ext_SPPB remained significant in men ($\beta$=-0.60, *p*=0.002), but not in women ($\beta$=-0.30, *p*=0.08) ≥ 65 years. The significant associations found between folate and HGS_ht_ in all women, and iron and _ext_SPPB in all men, were no longer significant in older adults (Q2 vs Q1 $\beta$=0.26, *p*=0.38, $\beta$=0.0041, *p*=0.12, respectively).

**References**

[1] NIH, Nutrient Recommendations and Databases, 2023. [cited 1st June 2023]. Available from: https://ods.od.nih.gov/HealthInformation/nutrientrecommendations.aspx.

[2] T.C. Cheng, S.H. Huang, C.L. Kao, P.C. Hsu, Muscle Wasting in Chronic Kidney Disease: Mechanism and Clinical Implications-A Narrative Review, Int. J. Mol. Sci. 23 (11) (2022) 6047, https://doi.org/10.3390/ijms23116047.

[3] J. Portoles, L. Martin, J.J. Broseta, A. Cases, Anemia in Chronic Kidney Disease: From Pathophysiology and Current Treatments, to Future Agents, Fronti. Med. (Lausanne) 8 (2021) 642296, https://doi.org/10.3389/fmed.2021.642296.

[4] M. Hassanein, T. Shafi, Assessment of glycemia in chronic kidney disease, BMC Med. 20 (1) (2022) 117, https://doi.org/10.1186/s12916-022-02316-1.

[5] C.S. Kim, S.W. Kim, Vitamin D and chronic kidney disease, Korean J. Intern. Med. 29 (4) (2014) 416-27, https://doi.org/10.3904/kjim.2014.29.4.416.

[6] G.M. McMahon, S. Hwang, R.M. Tanner, P.F. Jacques, J. Selhub, P. Muntner, et al., The association between vitamin B12, albuminuria and reduced kidney function: an observational cohort study, BMC Nephrol. 16 (2015) 7, https://doi.org/10.1186/1471-2369-16-7.

[7] K.M. Hill Gallant, D.M. Spiegel, Calcium Balance in Chronic Kidney Disease, Curr. Osteoporos. Rep. 15 (3) (2017) 214-221, https://doi.org/10.1007/s11914-017-0368-x.

[8] T. Dhondup, Q. Qian, Acid-Base and Electrolyte Disorders in Patients with and without Chronic Kidney Disease: An Update, Kidney Dis. (Basel) 3 (4) (2017) 136-148, https://doi.org/10.1159/000479968.

[9] G. Tomasz, W. Ewa, M. Jolanta, Biomarkers of iron metabolism in chronic kidney disease, Int. Urol. Nephrol. 53 (5) (2021) 935-944, https://doi.org/10.1007/s11255-020-02663-z.

[10] A. Wang, L.F. Yeung, N. Rios Burrows, C.E. Rose, Z. Fazili, C.M. Pfeiffer, et al., Reduced Kidney Function Is Associated with Increasing Red Blood Cell Folate Concentration and Changes in Folate Form Distributions (NHANES 2011-2018), Nutrients 14 (5) (2022) 1054, https://doi.org/10.3390/nu14051054.

[11] P.S. Suchdev, S.M. Namaste, G.J. Aaron, D.J. Raiten, K.H. Brown, R. Flores-Ayala, et al., Overview of the Biomarkers Reflecting Inflammation and Nutritional Determinants of Anemia (BRINDA) Project, Adv. Nutr. 7 (2) (2016) 349-56, https://doi.org/10.3945/an.115.010215.

[12] G.A. Ten Hoor, G. Plasqui, A. Schols, G. Kok, A Benefit of Being Heavier Is Being Strong: a Cross-Sectional Study in Young Adults, Sports Med. Open 4 (1) (2018) 12, https://doi.org/10.1186/s40798-018-0125-4.

[13] C. Liu, K.Y. Cheng, X. Tong, W.H. Cheung, S.K. Chow, S.W. Law, et al., The role of obesity in sarcopenia and the optimal body composition to prevent against sarcopenia and obesity, Front. Endocrinol. (Lausanne) 14 (2023) 1077255, https://doi.org/10.3389/fendo.2023.1077255.

[14] S.B. Heymsfield, P. Hwaung, F. Ferreyro-Bravo, M. Heo, D.M. Thomas, J.M. Schuna, Jr., Scaling of adult human bone and skeletal muscle mass to height in the US population, Am. J. Hum. Biol. 31 (4) (2019) e23252, https://doi.org/10.1002/ajhb.23252.

[15] D.J. Tomlinson, R.M. Erskine, C.I. Morse, K. Winwood, G. Onambele-Pearson, The impact of obesity on skeletal muscle strength and structure through adolescence to old age, Biogerontology 17 (3) (2016) 467-83, https://doi.org/10.1007/s10522-015-9626-4.

[16] R. Hardy, R. Cooper, A. Aihie Sayer, Y. Ben-Shlomo, C. Cooper, I.J. Deary, et al., Body mass index, muscle strength and physical performance in older adults from eight cohort studies: the HALCyon programme, PLoS One 8 (2) (2013) e56483, https://doi.org/10.1371/journal.pone.0056483.

**Supplementary Table 1** Proportion of participants with biomarker concentrations within, above or below normal range.

| **Measurement** | **Normal Range** | **N (%) below**  **normal range** | **N (%) within normal range** | **N (%) above**  **normal range** |
| --- | --- | --- | --- | --- |
|  |  |  |  |  |
| Haemoglobin (g/dL) |  |  |  |  |
| Women | 11.0 – 14.5 | 31 (3.5) | 796 (91.1) | 47 (5.4) |
| Men | 12.5 – 16.5 | 117 (13.8) | 720 (84.7) | 13 (1.5) |
| Ferritin (ng/mL) |  |  |  |  |
| Women | 5 – 148 | 5 (0.6) | 728 (89.4) | 81 (10.0) |
| Men | 28 – 365 | 57 (7.1) | 717 (89.6) | 26 (3.3) |
| Albumin (g/dL) |  |  |  |  |
| Women | 3.5 – 5.0 | 146 (16.4) | 745 (83.6) | 0 (0) |
| Men | 3.5 – 5.0 | 112 (12.9) | 758 (87.1) | 0 (0) |
| Creatinine (mg/dL) |  |  |  |  |
| Women | 0.52 – 1.04 | 10 (1.1) | 792 (89.2) | 86 (9.7) |
| Men | 0.66 – 1.50 | 10 (1.1) | 801 (92.2) | 58 (6.7) |
| HbA1c (%) |  |  |  |  |
| Women | 4.8 – 6.0 | 7 (0.9) | 585 (79.3) | 146 (19.8) |
| Men | 4.8 – 6.0 | 15 (2.2) | 509 (75.3) | 152 (22.5) |
| Vitamin D (ng/mL) |  |  |  |  |
| Women | 30 – 100 | 227 (31.1) | 502 (68.8) | 1 (0.1) |
| Men | 30 – 100 | 267 (39.6) | 408 (60.4) | 0 (0) |
| Vitamin B12 (pg/mL) |  |  |  |  |
| Women | 254 – 1320 | 23 (2.9) | 728 (92.3) | 38 (4.8) |
| Men | 254 – 1320 | 33 (4.2) | 726 (92.2) | 28 (3.6) |
| Folate (ng/mL) |  |  |  |  |
| Women | 2.8 – 14 | 0 (0) | 171 (21.3) | 631 (78.7) |
| Men | 2.8 – 14 | 0 (0) | 201 (25.7) | 582 (74.3) |
| Magnesium (mg/dL) |  |  |  |  |
| Women | 1.6 – 2.3 | 8 (1.0) | 730 (89.7) | 76 (9.3) |
| Men | 1.6 – 2.3 | 9 (1.1) | 711 (88.8) | 81 (10.1) |
| Calcium (mg/dL) |  |  |  |  |
| Women | 8.4 – 10.2 | 46 (5.2) | 833 (93.5) | 12 (1.3) |
| Men | 8.4 – 10.2 | 58 (6.7) | 807 (92.7) | 5 (0.6) |
| Potassium (mmol/L) |  |  |  |  |
| Women | 3.5 – 5.1 | 25 (2.8) | 860 (96.5) | 6 (0.7) |
| Men | 3.5 – 5.1 | 19 (2.2) | 839 (96.4) | 12 (1.4) |
| Iron ($\mu$g/dL) |  |  |  |  |
| Women | 37 – 170 | 19 (2.3) | 786 (96.6) | 9 (1.1) |
| Men | 49 – 181 | 45 (5.6) | 744 (93.4) | 8 (1.0) |

**Supplementary Table 2** Quintiles of serum folate in women (left) and men (right).

|  |  | **Women** | | | |  | **Men** | | | |
| --- | --- | --- | --- | --- | --- | --- | --- | --- | --- | --- |
| **Folate** |  | ***n*** | **Mean** | **Median** | **Range** |  | ***n*** | **Mean** | **Median** | **Range** |
| Q1 |  | 159 | 10.0 | 10.2 | 3.9 – 13.4 |  | 154 | 9.5 | 10.2 | 4.0 – 12.7 |
| Q2 |  | 161 | 16.2 | 16.3 | 13.5 – 18.3 |  | 155 | 15.0 | 15.0 | 12.8 – 17.0 |
| Q3 |  | 160 | 20.5 | 20.0 | 18.4 – 23.7 |  | 159 | 19.0 | 19.0 | 17.1 – 21.2 |
| Q4 |  | 161 | 28.4 | 28.5 | 23.8 – 33.1 |  | 158 | 25.6 | 25.5 | 21.3 – 30.3 |
| Q5 |  | 161 | 46.1 | 43.8 | 33.5 – 105.0 |  | 157 | 40.9 | 36.6 | 30.4 – 98.1 |

**Supplementary Table 3** Proportion of participants using dietary supplements or with missing (unknown) dietary supplement use information.

| **Supplement Use *n* (%)** | **All** | **Women** | **Men** |
| --- | --- | --- | --- |
| Vitamin D supplement use  None  < 14.5 mg/day  $\geq$14.5 mg/day  Unknown | 145 (8.2)  861 (48.9)  119 (6.8)  636 (36.1) | 59 (6.6)  474 (53.2)  77 (8.7)  281 (31.5) | 86 (9.9)  387 (44.5)  42 (4.8)  355 (40.8) |
| B12 supplement use  None  < 2.4$\mu$g/day  $\geq$ 2.4$\mu$g/day  Unknown | 191 (10.9)  632 (35.9)  302 (17.1)  636 (36.1) | 84 (9.4)  367 (41.2)  159 (17.9)  281 (31.5) | 107 (12.3)  265 (30.5)  143 (16.4)  355 (40.8) |
| Folate supplement use  None  < 400$\mu$g/day  $\geq$ 400$\mu$g/day  Unknown | 196 (11.1)  760 (43.2)  169 (9.6)  636 (36.1) | 87 (9.8)  434 (48.7)  89 (10.0)  281 (31.5) | 109 (12.5)  326 (37.5)  80 (9.2)  355 (40.8) |
| Magnesium supplement use  None  < 400mg/day  $\geq$400mg/day  Unknown | 195 (11.1)  861 (48.9)  69 (3.9)  636 (36.1) | 86 (9.7)  481 (54.0)  43 (4.8)  281 (31.5) | 109 (12.5)  380 (43.7)  26 (3.0)  355 (40.8) |
| Potassium supplement use  No  Yes  Unknown | 206 (11.7)  919 (52.2)  636 (36.1) | 91 (10.2)  519 (58.3)  281 (31.5) | 115 (13.2)  400 (46.0)  355 (40.8) |
| Calcium supplement use  None  < 1000mg/day  $\geq$ 1000mg/day  Unknown | 142 (8.1)  861 (48.9)  122 (6.9)  636 (36.1) | 57 (6.4)  478 (53.7)  75 (8.4)  281 (31.5) | 85 (9.8)  383 (44.0)  47 (5.4)  355 (40.8) |
| Iron supplement use  None  < 8mg/day  $\geq$8mg/day  Unknown | 209 (11.9)  802 (45.5)  114 (6.5)  636 (36.1) | 96 (10.8)  459 (51.5)  55 (6.2)  281 (31.5) | 113 (13.0)  343 (39.4)  59 (6.8)  355 (40.8) |

**Supplementary Table 4** Associations between clinical biomarkers and measures of lean mass in women.^1^

|  | **Women** | | | | | | | | | |
| --- | --- | --- | --- | --- | --- | --- | --- | --- | --- | --- |
|  |  | **Model 1***^2^* | | | |  | **Model 2***^3^* | | | |
| **ALM,***^4^* **kg** | ***n*** | $\boldsymbol{\beta\pm}$**SE** | | **R^2^** | ***p*** |  | $\boldsymbol{\beta}$ $\boldsymbol{\pm}$ **SE** | **R^2^** | | ***p*** |
| Haemoglobin, g/dL | 704 | -0.20 $\pm$ 0.10 | | 0.005 | 0.06 |  | -0.095 $\pm$ 0.076 | 0.54 | | 0.21 |
| Ferritin,*^5^* ng/mL | 706 | 0.00051 $\pm$0.0016 | | 0.0002 | 0.74 |  | 0.0020 $\pm$ 0.0011 | 0.54 | | 0.07 |
| Albumin, g/dL | 709 | -0.44 $\pm$ 0.31 | | 0.003 | 0.16 |  | -0.64 $\pm$ 0.23 | 0.54 | | 0.005* |
| Creatinine,*^6^* mg/dL | 709 | 0.0064 $\pm$ 0.55 | | <0.0001 | 0.99 |  | 0.78 $\pm$ 0.39 | 0.54 | | 0.045* |
|  |  |  |  |  |  |  |  |  |  |  |
| **ALM_ht,_***^7^* **kg/m^2^** |  |  |  |  |  |  |  |  |  |  |
| Haemoglobin, g/dL | 704 | -0.10 $\pm$0.03 | | 0.01 | 0.002* |  | -0.057 $\pm$0.027 | 0.42 | | 0.034* |
| Ferritin,*^5^* ng/mL | 706 | 0.00073 $\pm$0.00049 | | 0.003 | 0.14 |  | 0.00058 $\pm$0.00039 | 0.43 | | 0.13 |
| Albumin, g/dL | 709 | -0.25 $\pm$0.10 | | 0.009 | 0.011* |  | -0.26 $\pm$0.08 | 0.43 | | 0.001* |
| Creatinine,*^6^* mg/dL | 709 | 0.18 $\pm$0.17 | | 0.002 | 0.31 |  | 0.19 $\pm$0.14 | 0.42 | | 0.16 |
|  |  |  |  |  |  |  |  |  |  |  |
| **FFM%***^7^* |  |  |  |  |  |  |  |  |  |  |
| Haemoglobin, g/dL | 704 | -0.0085 $\pm$0.30 | | <0.0001 | 0.98 |  | -0.20 $\pm$0.18 | 0.71 | | 0.26 |
| Ferritin,*^5^* ng/mL | 706 | -0.012 $\pm$0.004 | | 0.01 | 0.007* |  | 0.0029 $\pm$0.0025 | 0.71 | | 0.25 |
| Albumin, g/dL | 709 | 1.71 $\pm$0.91 | | 0.005 | 0.06 |  | -0.60 $\pm$0.52 | 0.71 | | 0.25 |
| Creatinine,*^6^* mg/dL | 709 | -0.44 $\pm$1.59 | | 0.0001 | 0.78 |  | 1.79 $\pm$0.89 | 0.71 | | 0.044* |

*^1^* Data are presented as regression coefficients ($\beta$) $\pm$ standard error (SE). * denotes statistical significance. ALM: appendicular lean mass; ALM_ht_: height-adjusted ALM; FFM%: fat-free mass as a percentage of body weight.

*^2^* Model 1 is unadjusted.

*^3^* Model 2 is adjusted for age (years), smoking status (never, former or current), race (White American, Black American, Other) physical activity level (not active, moderately active, active, highly active) and estimated glomerular filtration rate (ml/min/1.73m^2^) for all outcomes.

*^4^* Models containing ALM are additionally adjusted for height (cm) and appendicular fat mass (kg).

*^5^* Models containing ferritin were additionally adjusted for C-reactive protein category ($\leq$ 5$\mu$g/mL, $>$5 $\mu$g/mL).

*^6^* Models containing creatinine were not adjusted for eGFR due to collinearity.

*^7^* ALM_ht_ and FFM% models are additionally adjusted for body mass index (kg/m^2^).

**Supplementary Table 5** Associations between clinical biomarkers and measures of lean mass in men.^1^

|  | **Men** | | | | | | | | | | |
| --- | --- | --- | --- | --- | --- | --- | --- | --- | --- | --- | --- |
|  |  | **Model 1***^2^* | | | |  | **Model 2***^3^* | | | | |
| **ALM,***^4^* **kg** | ***n*** | $\boldsymbol{\beta}\pm$**SE** | | **R^2^** | ***p*** |  | $\boldsymbol{\beta}\pm$**SE** | **R^2^** | | | ***p*** |
| Haemoglobin, g/dL | 651 | 0.92 $\pm$0.12 | | 0.09 | <0.001* |  | 0.20 $\pm$0.09 | 0.60 | | | 0.021* |
| Ferritin,*^5^* ng/mL | 656 | 0.0052 $\pm$0.0016 | | 0.02 | 0.002* |  | 0.00021 $\pm$0.0011 | 0.60 | | | 0.85 |
| Albumin, g/dL | 658 | 1.79 $\pm$0.47 | | 0.02 | <0.001* |  | -0.94 $\pm$0.32 | 0.60 | | | 0.004* |
| Creatinine,*^6^* mg/dL | 658 | -0.87 $\pm$0.61 | | 0.003 | 0.16 |  | 1.28 $\pm$0.41 | 0.59 | | | 0.002* |
|  |  |  |  |  |  |  |  | |  |  |  |
| **ALM_ht,_***^7^* **kg/m^2^** |  |  |  |  |  |  |  | |  |  |  |
| Haemoglobin, g/dL | 650 | 0.22 $\pm$0.03 | | 0.07 | <0.001* |  | 0.026 $\pm$0.027 | 0.46 | | | 0.35 |
| Ferritin,*^5^* ng/mL | 655 | 0.0011 $\pm$0.0004 | | 0.01 | 0.013* |  | -0.00033 $\pm$0.00032 | 0.46 | | | 0.30 |
| Albumin, g/dL | 657 | 0.33 $\pm$0.12 | | 0.01 | 0.007* |  | -0.24 $\pm$0.10 | 0.47 | | | 0.017* |
| Creatinine,*^6^* mg/dL | 657 | -0.016 $\pm$0.16 | | <0.0001 | 0.92 |  | 0.34 $\pm$0.12 | 0.46 | | | 0.006* |
|  |  |  |  |  |  |  |  | |  |  |  |
| **FFM%***^7^* |  |  |  |  |  |  |  | |  |  |  |
| Haemoglobin, g/dL | 650 | -0.51 $\pm$0.23 | | 0.008 | 0.028* |  | -0.072 $\pm$0.17 | 0.62 | | | 0.66 |
| Ferritin,*^5^* ng/mL | 655 | -0.012 $\pm$0.003 | | 0.02 | <0.001* |  | -0.0034 $\pm$0.0020 | 0.62 | | | 0.08 |
| Albumin, g/dL | 657 | 1.51 $\pm$0.89 | | 0.004 | 0.09 |  | -0.88 $\pm$0.60 | 0.62 | | | 0.14 |
| Creatinine,*^6^* mg/dL | 657 | -0.50 $\pm$1.16 | | 0.0003 | 0.67 |  | 2.64 $\pm$0.75 | 0.62 | | | <0.001* |

*^1^* Data are presented as regression coefficients ($\beta$) $\pm$ standard error (SE). * denotes statistical significance. ALM: appendicular lean mass; ALM_ht_: height-adjusted ALM; FFM%: fat-free mass as a percentage of body weight.

*^2^* Model 1 is unadjusted.

*^3^* Model 2 is adjusted for age (years), smoking status (never, former or current), race (White American, Black American, Other) physical activity level (not active, moderately active, active, highly active) and estimated glomerular filtration rate (ml/min/1.73m^2^) for all outcomes.

*^4^* Models containing ALM are additionally adjusted for height (cm) and appendicular fat mass (kg).

*^5^* Models containing ferritin were additionally adjusted for C-reactive protein category ($\leq$ 5$\mu$g/mL, $>$5 $\mu$g/mL).

*^6^* Models containing creatinine were not adjusted for eGFR due to collinearity.

*^7^* ALM_ht_ and FFM% models are additionally adjusted for body mass index (kg/m^2^).

**Supplementary Table 6** Associations between clinical biomarkers and muscle strength or function in women.^1^

|  | **Women** | | | | | | | | | | |
| --- | --- | --- | --- | --- | --- | --- | --- | --- | --- | --- | --- |
|  |  | **Model 1***^3^* | | | |  | **Model 2***^4^* | | | | |
| **Extended SPPB Score*^2^*** | ***n*** | $\boldsymbol{\beta}\pm$**SE** | | **R^2^** | ***p*** |  | $\boldsymbol{\beta}\pm$**SE** | **R^2^** | | | ***p*** |
| Haemoglobin, g/dL | 704 | 0.37 $\pm$0.10 | | 0.02 | <0.001* |  | 0.15 $\pm$0.06 | 0.63 | | | 0.019* |
| Ferritin,*^5^* ng/mL | 696 | -0.0068 $\pm$0.0014 | | 0.03 | <0.001* |  | -0.00079 $\pm$0.00093 | 0.62 | | | 0.40 |
| Albumin, g/dL | 710 | 1.00 $\pm$0.29 | | 0.02 | 0.001* |  | -0.50 $\pm$0.19 | 0.63 | | | 0.008* |
| Creatinine*^6^* mg/dL | 710 | -2.56 $\pm$0.47 | | 0.04 | <0.001* |  | -0.43 $\pm$0.30 | 0.63 | | | 0.15 |
|  |  |  |  |  |  |  |  | |  |  |  |
| **HGS_ht_, kg/m^2^** |  |  |  |  |  |  |  | |  |  |  |
| Haemoglobin, g/dL | 777 | 0.047 $\pm$0.083 | | 0.0004 | 0.58 |  | 0.079 $\pm$0.069 | 0.38 | | | 0.26 |
| Ferritin,*^5^* ng/mL | 719 | -0.0017 $\pm$0.0013 | | 0.002 | 0.19 |  | 0.0013 $\pm$0.0011 | 0.36 | | | 0.25 |
| Albumin, g/dL | 787 | 0.82 $\pm$0.24 | | 0.01 | 0.001* |  | 0.30 $\pm$0.20 | 0.37 | | | 0.14 |
| Creatinine,*^6^* mg/dL | 787 | -0.55 $\pm$0.40 | | 0.003 | 0.16 |  | 0.54 $\pm$0.32 | 0.37 | | | 0.10 |
|  |  |  |  |  |  |  |  | |  |  |  |
| **KEC_ht_, Nm/m^2^** | | |  |  |  |  |  | |  |  |  |
| Haemoglobin, g/dL | 550 | 0.13 $\pm$0.44 | | 0.0002 | 0.77 |  | 0.27 $\pm$0.36 | 0.39 | | | 0.46 |
| Ferritin,*^5^* ng/mL | 551 | -0.0072 $\pm$0.0067 | | 0.002 | 0.28 |  | 0.012 $\pm$0.005 | 0.40 | | | 0.031* |
| Albumin, g/dL | 552 | 7.45 $\pm$1.42 | | 0.05 | <0.001* |  | 1.89 $\pm$1.20 | 0.39 | | | 0.12 |
| Creatinine,*^6^* mg/dL | 552 | -2.61 $\pm$2.91 | | 0.002 | 0.37 |  | 1.42 $\pm$2.40 | 0.39 | | | 0.56 |
|  |  |  |  |  |  |  |  | |  |  |  |
| **KEI_ht_, Nm/m^2^** | | |  |  |  |  |  | |  |  |  |
| Haemoglobin, g/dL | 545 | 0.089 $\pm$0.49 | | 0.0001 | 0.86 |  | 0.43 $\pm$0.41 | 0.39 | | | 0.29 |
| Ferritin,*^5^* ng/mL | 546 | -0.0054 $\pm$0.0075 | | 0.001 | 0.47 |  | 0.013 $\pm$0.006 | 0.40 | | | 0.031* |
| Albumin, g/dL | 547 | 8.39 $\pm$1.59 | | 0.05 | <0.001* |  | 2.89 $\pm$1.34 | 0.39 | | | 0.032* |
| Creatinine,*^6^* mg/dL | 547 | -0.89 $\pm$3.25 | | 0.0001 | 0.79 |  | 2.27 $\pm$2.68 | 0.39 | | | 0.40 |

*^1^* Data are presented as regression coefficients ($\beta$) $\pm$ standard error (SE). * denotes statistical significance. HGS_ht_: height-adjusted hand grip strength; KEC_ht_: height-adjusted knee extension concentric strength; KEI_ht_: height-adjusted knee extension isometric strength.
*^2^* Extended short physical performance battery (SPPB) score was transformed using a Box-Cox transformation in all models.

*^3^* Model 1 is unadjusted.

*^4^* Model 2 is adjusted for age (years), body mass index (kg/m^2^) smoking status (never, former or current), race (White American, Black American, Other), physical activity level (not active, moderately active, active, highly active) and estimated glomerular filtration rate (ml/min/1.73m^2^).

*^5^* Models containing ferritin were additionally adjusted for C-reactive protein category ($\leq$ 5$\mu$g/mL, $>$5 $\mu$g/mL).

*^6^* Models containing creatinine were not adjusted for eGFR due to collinearity.

**Supplementary Table 7** Associations between clinical biomarkers and muscle strength or function in men.^1^

|  | **Men** | | | | | | | | | |
| --- | --- | --- | --- | --- | --- | --- | --- | --- | --- | --- |
|  |  | **Model 1***^3^* | | | |  | **Model 2***^4^* | | | |
| **Extended SPPB Score***^2^* | ***n*** | $\boldsymbol{\beta}$ $\pm$**SE** | | **R^2^** | ***p*** |  | $\boldsymbol{\beta}$ $\pm$**SE** | **R^2^** | | ***p*** |
| Haemoglobin, g/dL | 633 | 0.68 $\pm$0.07 | | 0.13 | <0.001* |  | 0.13 $\pm$0.06 | 0.56 | | 0.024* |
| Ferritin,*^5^* ng/mL | 635 | 0.0018 $\pm$0.0010 | | 0.005 | 0.07 |  | -0.00043 $\pm$0.00068 | 0.56 | | 0.53 |
| Albumin, g/dL | 641 | 1.52 $\pm$0.28 | | 0.04 | <0.001* |  | -0.60 $\pm$0.20 | 0.56 | | 0.003* |
| Creatinine,*^6^* mg/dL | 641 | -2.05 $\pm$0.37 | | 0.05 | <0.001* |  | -0.36 $\pm$0.26 | 0.56 | | 0.16 |
|  |  |  |  |  |  |  |  |  |  |  |
| **HGS_ht_, kg/m^2^** |  |  |  |  |  |  |  |  |  |  |
| Haemoglobin, g/dL | 723 | 0.89 $\pm$0.08 | | 0.14 | <0.001* |  | 0.25 $\pm$0.07 | 0.46 | | 0.001* |
| Ferritin,*^5^* ng/mL | 677 | 0.0031 $\pm$0.0012 | | 0.01 | 0.01* |  | -0.000032 $\pm$0.00093 | 0.43 | | 0.97 |
| Albumin, g/dL | 736 | 1.86 $\pm$0.33 | | 0.04 | <0.001* |  | -0.12 $\pm$0.27 | 0.45 | | 0.66 |
| Creatinine,*^6^* mg/dL | 736 | -1.46 $\pm$0.45 | | 0.01 | 0.001* |  | 0.16 $\pm$0.35 | 0.45 | | 0.64 |
|  |  |  |  |  |  |  |  |  |  |  |
| **KEC_ht_, Nm/m^2^** | | |  |  |  |  |  |  |  |  |
| Haemoglobin, g/dL | 484 | 3.19 $\pm$0.48 | | 0.08 | <0.001* |  | 0.45 $\pm$0.44 | 0.42 | | 0.31 |
| Ferritin,*^5^* ng/mL | 488 | 0.014 $\pm$0.007 | | 0.009 | 0.034* |  | -0.0015 $\pm$0.0054 | 0.42 | | 0.78 |
| Albumin, g/dL | 490 | 8.95 $\pm$1.95 | | 0.04 | <0.001* |  | -0.99 $\pm$1.65 | 0.41 | | 0.55 |
| Creatinine,*^6^* mg/dL | 490 | -6.49 $\pm$2.88 | | 0.01 | 0.025* |  | 1.97 $\pm$2.32 | 0.41 | | 0.40 |
|  |  |  |  |  |  |  |  |  |  |  |
| **KEI_ht_, Nm/m^2^** | | |  |  |  |  |  |  |  |  |
| Haemoglobin, g/dL | 481 | 3.57 $\pm$0.55 | | 0.08 | <0.001* |  | 0.65 $\pm$0.49 | 0.42 | | 0.19 |
| Ferritin,*^5^* ng/mL | 485 | 0.019 $\pm$0.008 | | 0.01 | 0.015* |  | -0.00080 $\pm$0.0061 | 0.42 | | 0.90 |
| Albumin, g/dL | 487 | 11.61 $\pm$2.20 | | 0.05 | <0.001* |  | 1.14 $\pm$1.88 | 0.42 | | 0.55 |
| Creatinine,*^6^* mg/dL | 487 | -5.51 $\pm$3.29 | | 0.006 | 0.10 |  | 2.69 $\pm$2.64 | 0.41 | | 0.31 |

*^1^* Data are presented as regression coefficients ($\beta$) $\pm$ standard error (SE). * denotes statistical significance. HGS_ht_: height-adjusted hand grip strength; KEC_ht_: height-adjusted knee extension concentric strength; KEI_ht_: height-adjusted knee extension isometric strength.
*^2^* Extended short physical performance battery (SPPB) score was transformed using a Box-Cox transformation in all models.

*^3^* Model 1 is unadjusted.

*^4^* Model 2 is adjusted for age (years), body mass index (kg/m^2^) smoking status (never, former or current), race (White American, Black American, Other), physical activity level (not active, moderately active, active, highly active) and estimated glomerular filtration rate (ml/min/1.73m^2^).

*^5^* Models containing ferritin were additionally adjusted for C-reactive protein category ($\leq$ 5$\mu$g/mL, $>$5 $\mu$g/mL).

*^6^* Models containing creatinine were not adjusted for eGFR due to collinearity.

**Supplementary Table 8** Associations between nutritional biomarkers and measures of lean mass in women.*^1^*

|  | **Women** | | | | | | | | | | | | | | | | |
| --- | --- | --- | --- | --- | --- | --- | --- | --- | --- | --- | --- | --- | --- | --- | --- | --- | --- |
|  |  | **Model 1***^2^* | | | |  | **Model 2***^3^* | | | |  | **Model 3***^4^* | | | | | |
| **ALM,**^5^ **kg** | ***n*** | $\boldsymbol{\beta}$ $\pm$**SE** | | **R^2^** | ***p*** |  | $\boldsymbol{\beta}$ $\pm$**SE** | | **R^2^** | ***p*** |  | $\boldsymbol{\beta}$ $\pm$**SE** | **R^2^** | | ***p*** | | |
| Vitamin D, ng/mL | 653 | -0.021 $\pm$0.008 | | 0.01 | 0.011* |  | -0.00045 $\pm$0.0057 | | 0.54 | 0.94 |  | 0.00021 $\pm$0.0057 | 0.55 | | 0.97 | | |
| Vitamin B12, pg/mL | 686 | -0.00034 $\pm$0.00032 | | 0.002 | 0.30 |  | 0.00025 $\pm$0.00023 | | 0.54 | 0.28 |  | 0.00027 $\pm$0.00023 | 0.54 | | 0.24 | | |
| Magnesium, mg/dL | 709 | -0.63 $\pm$0.54 | | 0.002 | 0.25 |  | -0.21 $\pm$0.38 | | 0.54 | 0.59 |  | -0.23 $\pm$0.38 | 0.54 | | 0.55 | | |
| Potassium, mmol/L | 709 | -0.26 $\pm$0.37 | | 0.0007 | 0.48 |  | 0.20 $\pm$0.25 | | 0.54 | 0.44 |  | 0.20 $\pm$0.26 | 0.54 | | 0.44 | | |
| Calcium, mg/dL | 709 | -0.45 $\pm$0.24 | | 0.005 | 0.06 |  | -0.40 $\pm$0.17 | | 0.54 | 0.018* |  | -0.37 $\pm$0.17 | 0.54 | | 0.031* | | |
| Iron, $\mu$g/dL | 709 | -0.0092 $\pm$0.0038 | | 0.008 | 0.015* |  | 0.00059 $\pm$0.0027 | | 0.54 | 0.83 |  | 0.00051 $\pm$0.0027 | 0.54 | | 0.85 | | |
|  |  |  |  |  |  |  |  |  |  |  |  |  | |  | |  |  |
| **ALM_ht_,***^6^* **kg/m^2^** |  |  |  |  |  |  |  |  |  |  |  |  | |  | |  |  |
| Vitamin D, ng/mL | 653 | -0.0061 $\pm$0.0026 | | 0.009 | 0.018* |  | 0.0013 $\pm$0.0020 | | 0.43 | 0.52 |  | 0.0015 $\pm$0.0020 | 0.43 | | 0.46 | | |
| Vitamin B12, pg/mL | 686 | 0.000012 $\pm$0.00010 | | <0.0001 | 0.91 |  | 0.00016 $\pm$0.00008 | | 0.43 | 0.049* |  | 0.00017 $\pm$0.00008 | 0.43 | | 0.035* | | |
| Magnesium, mg/dL | 709 | -0.13 $\pm$0.17 | | 0.0008 | 0.45 |  | 0.015 $\pm$0.13 | | 0.42 | 0.91 |  | 0.0084 $\pm$0.14 | 0.42 | | 0.95 | | |
| Potassium, mmol/L | 709 | -0.033 $\pm$0.12 | | 0.0001 | 0.78 |  | 0.049 $\pm$0.090 | | 0.42 | 0.58 |  | 0.048 $\pm$0.091 | 0.42 | | 0.60 | | |
| Calcium, mg/dL | 709 | -0.12 $\pm$0.08 | | 0.004 | 0.10 |  | -0.17 $\pm$0.06 | | 0.43 | 0.003* |  | -0.16 $\pm$0.06 | 0.43 | | 0.008* | | |
| Iron, $\mu$g/dL | 709 | -0.0038 $\pm$0.0012 | | 0.01 | 0.001* |  | 0.000070 $\pm$0.00094 | | 0.42 | 0.94 |  | 0.000023 $\pm$0.00094 | 0.42 | | 0.98 | | |
|  |  |  |  |  |  |  |  |  |  |  |  |  | |  | |  |  |
| **FFM%***^6^* |  |  |  |  |  |  |  |  |  |  |  |  | |  | |  |  |
| Vitamin D, ng/mL | 653 | 0.075 $\pm$0.023 | | 0.02 | 0.001* |  | 0.0083 $\pm$0.013 | | 0.70 | 0.53 |  | 0.0083 $\pm$0.013 | 0.70 | | 0.53 | | |
| Vitamin B12, pg/mL | 686 | 0.0027 $\pm$0.0009 | | 0.01 | 0.004* |  | 0.0010 $\pm$0.0005 | | 0.71 | 0.051 |  | 0.00097 $\pm$0.00053 | 0.71 | | 0.07 | | |
| Magnesium, mg/dL | 709 | 3.09 $\pm$1.57 | | 0.006 | 0.049* |  | -1.01 $\pm$0.88 | | 0.71 | 0.25 |  | -1.09 $\pm$0.89 | 0.71 | | 0.22 | | |
| Potassium, mmol/L | 709 | 0.56 $\pm$1.07 | | 0.0004 | 0.60 |  | 0.49 $\pm$0.59 | | 0.71 | 0.41 |  | 0.53 $\pm$0.59 | 0.71 | | 0.37 | | |
| Calcium, mg/dL | 709 | -1.48 $\pm$0.69 | | 0.007 | 0.032* |  | -0.50 $\pm$0.38 | | 0.71 | 0.20 |  | -0.51 $\pm$0.39 | 0.71 | | 0.20 | | |
| Iron, $\mu$g/dL | 709 | 0.034 $\pm$0.011 | | 0.01 | 0.002* |  | 0.0025 $\pm$0.0062 | | 0.71 | 0.69 |  | 0.0024 $\pm$0.0062 | 0.71 | | 0.70 | | |

*^1^* Data are presented as regression coefficients ($\beta$) $\pm$ standard error (SE). * denotes statistical significance. ALM: appendicular lean mass; ALM_ht_: height-adjusted ALM; FFM%: fat-free mass as a percentage of body weight

*^2^* Model 1 is unadjusted.

*^3^* Model 2 for is adjusted for age (years), smoking status (never, former or current), race (White American, Black American, Other), physical activity level (not active, moderately active, active, highly active) and estimated glomerular filtration rate (ml/min/1.73m^2^) for all outcomes.

*^4^* Model 3 is additionally adjusted for dietary supplement use for the corresponding nutrient.

*^5^* ALM models are additionally adjusted for height (cm) and appendicular fat mass (kg).

*^6^* ALM_ht_ and FFM% models are additionally adjusted for body mass index (kg/m^2^).

**Supplementary Table 9** Associations between nutritional biomarkers and measures of lean mass in men.*^1^*

|  | **Men** | | | | | | | | | | | | | | | | |
| --- | --- | --- | --- | --- | --- | --- | --- | --- | --- | --- | --- | --- | --- | --- | --- | --- | --- |
|  |  | **Model 1***^2^* | | | |  | **Model 2***^3^* | | | |  | **Model 3***^4^* | | | | | |
| **ALM,***^5^* **kg** | ***n*** | $\boldsymbol{\beta}$ $\pm$**SE** | | **R^2^** | ***p*** |  | $\boldsymbol{\beta}$ $\pm$**SE** | | **R^2^** | ***p*** |  | $\boldsymbol{\beta}$ $\pm$**SE** | **R^2^** | | ***p*** | | |
| Vitamin D, ng/mL | 589 | -0.058 $\pm$0.014 | | 0.03 | <0.001* |  | -0.013 $\pm$0.010 | | 0.60 | 0.18 |  | -0.012 $\pm$0.010 | 0.60 | | 0.22 | | |
| Vitamin B12, pg/mL | 645 | -0.0013 $\pm$0.0006 | | 0.008 | 0.027* |  | -0.00044 $\pm$0.00037 | | 0.59 | 0.24 |  | -0.00036 $\pm$0.00038 | 0.59 | | 0.34 | | |
| Magnesium, mg/dL | 656 | -0.55 $\pm$0.77 | | 0.0008 | 0.47 |  | -0.31 $\pm$0.50 | | 0.60 | 0.53 |  | -0.28 $\pm$0.50 | 0.60 | | 0.58 | | |
| Potassium, mmol/L | 658 | -0.15 $\pm$0.52 | | 0.0001 | 0.78 |  | 0.62 $\pm$0.33 | | 0.60 | 0.06 |  | 0.57 $\pm$0.34 | 0.60 | | 0.09 | | |
| Calcium, mg/dL | 658 | -0.057 $\pm$0.36 | | <0.0001 | 0.88 |  | -0.62 $\pm$0.23 | | 0.60 | 0.008* |  | -0.60 $\pm$0.23 | 0.60 | | 0.011* | | |
| Iron, $\mu$g/dL | 656 | 0.013 $\pm$0.005 | | 0.009 | 0.013* |  | 0.0058 $\pm$0.0033 | | 0.60 | 0.08 |  | 0.0055 $\pm$0.0033 | 0.60 | | 0.10 | | |
|  |  |  |  |  |  |  |  |  |  |  |  |  | |  | |  |  |
| **ALM_ht_,***^6^* **kg/m^2^** |  |  |  |  |  |  |  |  |  |  |  |  | |  | |  |  |
| Vitamin D, ng/mL | 588 | -0.015 $\pm$0.004 | | 0.03 | <0.001* |  | -0.0012 $\pm$0.0030 | | 0.46 | 0.68 |  | -0.0010 $\pm$0.0031 | 0.45 | | 0.74 | | |
| Vitamin B12, pg/mL | 644 | -0.00029 $\pm$0.00015 | | 0.006 | 0.052 |  | -0.000019 $\pm$0.00011 | | 0.46 | 0.87 |  | -0.0000046 $\pm$0.00011 | 0.46 | | 0.97 | | |
| Magnesium, mg/dL | 655 | -0.16 $\pm$0.20 | | 0.001 | 0.42 |  | -0.058 $\pm$0.15 | | 0.46 | 0.70 |  | -0.049 $\pm$0.15 | 0.46 | | 0.75 | | |
| Potassium, mmol/L | 657 | 0.039 $\pm$0.14 | | 0.0001 | 0.77 |  | 0.15 $\pm$0.10 | | 0.46 | 0.13 |  | 0.14 $\pm$0.10 | 0.46 | | 0.16 | | |
| Calcium, mg/dL | 657 | -0.10 $\pm$0.09 | | 0.002 | 0.29 |  | -0.19 $\pm$0.07 | | 0.47 | 0.006* |  | -0.20 $\pm$0.07 | 0.47 | | 0.006* | | |
| Iron, $\mu$g/dL | 655 | 0.0035 $\pm$0.0013 | | 0.01 | 0.011* |  | 0.0022 $\pm$0.0010 | | 0.46 | 0.034* |  | 0.0021 $\pm$0.0010 | 0.46 | | 0.038* | | |
|  |  |  |  |  |  |  |  |  |  |  |  |  | |  | |  |  |
| **FFM%***^6^* |  |  |  |  |  |  |  |  |  |  |  |  | |  | |  |  |
| Vitamin D, ng/mL | 588 | 0.051 $\pm$0.028 | | 0.006 | 0.07 |  | -0.012 $\pm$0.018 | | 0.63 | 0.51 |  | -0.013 $\pm$0.018 | 0.63 | | 0.47 | | |
| Vitamin B12, pg/mL | 644 | 0.0020 $\pm$0.0011 | | 0.005 | 0.06 |  | -0.000078 $\pm$0.00068 | | 0.62 | 0.91 |  | -0.000076 $\pm$0.00069 | 0.62 | | 0.91 | | |
| Magnesium, mg/dL | 655 | 2.17 $\pm$1.45 | | 0.003 | 0.14 |  | -0.50 $\pm$0.92 | | 0.62 | 0.59 |  | -0.44 $\pm$0.92 | 0.62 | | 0.63 | | |
| Potassium, mmol/L | 657 | 0.45 $\pm$0.98 | | 0.0003 | 0.65 |  | 0.74 $\pm$0.61 | | 0.62 | 0.23 |  | 0.70 $\pm$0.62 | 0.62 | | 0.26 | | |
| Calcium, mg/dL | 657 | -0.24 $\pm$0.68 | | 0.0002 | 0.72 |  | -0.54 $\pm$0.43 | | 0.62 | 0.20 |  | -0.56 $\pm$0.43 | 0.62 | | 0.20 | | |
| Iron, $\mu$g/dL | 655 | 0.0029 $\pm$0.0097 | | 0.0001 | 0.76 |  | 0.0022 $\pm$0.0062 | | 0.62 | 0.72 |  | 0.0020 $\pm$0.0062 | 0.62 | | 0.75 | | |

*^1^* Data are presented as regression coefficients ($\beta$) $\pm$ standard error (SE). * denotes statistical significance. ALM: appendicular lean mass; ALM_ht_: height-adjusted ALM; FFM%: fat-free mass as a percentage of body weight

*^2^* Model 1 is unadjusted.

*^3^* Model 2 for is adjusted for age (years), smoking status (never, former or current), race (White American, Black American, Other), physical activity level (not active, moderately active, active, highly active) and estimated glomerular filtration rate (ml/min/1.73m^2^) for all outcomes.

*^4^* Model 3 is additionally adjusted for dietary supplement use for the corresponding nutrient.

*^5^* ALM models are additionally adjusted for height (cm) and appendicular fat mass (kg).

*^6^* ALM_ht_ and FFM% models are additionally adjusted for body mass index (kg/m^2^).

**Supplementary Table 10** Associations between serum folate and sarcopenic indices in women.^1^

|  | | **Women** | | | | | | | | | | | |
| --- | --- | --- | --- | --- | --- | --- | --- | --- | --- | --- | --- | --- | --- |
|  | | **Model 1***^2^* | | |  | **Model 2***^3^* | | |  | **Model 3***^4^* | | |  |
|  | | $\boldsymbol{\beta}$ $\pm$**SE** | ***p*** | **R^2^** |  | $\boldsymbol{\beta}$ $\pm$**SE** | ***p*** | **R^2^** |  | $\boldsymbol{\beta}$ $\pm$**SE** | ***p*** | **R^2^** |  |
| **ALM,**^5^ **kg** | |  |  |  |  |  |  |  |  |  |  |  |  |
| *n* = 703 | Q2 | -0.79 (0.34) | 0.02* | 0.03 |  | 0.11 (0.24) | 0.65 | 0.54 |  | 0.12 (0.24) | 0.63 | 0.54 |  |
| Q3 | | -0.71 (0.34) | 0.039* |  |  | 0.008 (0.24) | 0.97 |  |  | 0.023 (0.24) | 0.93 |  |  |
| Q4 | | -1.02 (0.34) | 0.003* |  |  | -0.14 (0.24) | 0.57 |  |  | -0.11 (0.24) | 0.67 |  |  |
| Q5 | | -1.50 (0.35) | <0.001* |  |  | -0.052 (0.25) | 0.83 |  |  | -0.031 (0.25) | 0.90 |  |  |
|  | |  |  |  |  |  |  |  |  |  |  |  |  |
| **ALM_ht_,***^6^* **kg/m^2^** | |  |  |  |  |  |  |  |  |  |  |  |  |
| *n* = 703 | Q2 | -0.22 (0.11) | 0.039* | 0.02 |  | 0.081 (0.084) | 0.34 | 0.42 |  | 0.087 (0.084) | 0.30 | 0.42 |  |
| Q3 | | -0.14 (0.11) | 0.20 |  |  | 0.014 (0.084) | 0.87 |  |  | 0.023 (0.085) | 0.78 |  |  |
| Q4 | | -0.29 (0.11) | 0.007* |  |  | -0.038 (0.085) | 0.65 |  |  | -0.019 (0.086) | 0.82 |  |  |
| Q5 | | -0.41 (0.11) | <0.001* |  |  | 0.0096 (0.087) | 0.91 |  |  | 0.023 (0.088) | 0.79 |  |  |
|  | |  |  |  |  |  |  |  |  |  |  |  |  |
| **FFM%***^6^* | |  |  |  |  |  |  |  |  |  |  |  |  |
| *n* = 703 | Q2 | 4.38 (0.98) | <0.001* | 0.03 |  | 1.23 (0.55) | 0.025* | 0.71 |  | 1.24 (0.55) | 0.025* | 0.71 |  |
| Q3 | | 2.15 (1.00) | 0.031* |  |  | 0.78 (0.55) | 0.16 |  |  | 0.80 (0.55) | 0.15 |  |  |
| Q4 | | 3.50 (0.99) | <0.001* |  |  | 1.27 (0.55) | 0.021* |  |  | 1.32 (0.56) | 0.019* |  |  |
| Q5 | | 3.15 (0.99) | 0.002* |  |  | 0.68 (0.57) | 0.23 |  |  | 0.72 (0.57) | 0.21 |  |  |
|  | |  |  |  |  |  |  |  |  |  |  |  |  |
| **Extended SPPB Score***^6,7^* | |  |  |  |  |  |  |  |  |  |  |  |  |
| *n* = 691 | Q2 | 0.94 (0.31) | 0.003* | 0.04 |  | 0.46 (0.20) | 0.022* | 0.62 |  | 0.41 (0.20) | 0.038* | 0.63 |  |
| Q3 | | 0.78 (0.32) | 0.015* |  |  | 0.39 (0.20) | 0.051 |  |  | 0.36 (0.20) | 0.07 |  |  |
| Q4 | | 0.65 (0.31) | 0.038* |  |  | 0.23 (0.20) | 0.25 |  |  | 0.19 (0.20) | 0.35 |  |  |
| Q5 | | -0.54 (0.32) | 0.09 |  |  | -0.11 (0.20) | 0.58 |  |  | -0.17 (0.20) | 0.40 |  |  |
|  | |  |  |  |  |  |  |  |  |  |  |  |  |
| **HGS_ht_,** *^6^* **kg/m^2^** | |  |  |  |  |  |  |  |  |  |  |  |  |
| *n* = 715 | Q2 | 0.37 (0.28) | 0.19 | 0.03 |  | 0.58 (0.23) | 0.014* | 0.36 |  | 0.56 (0.24) | 0.018* | 0.36 |  |
| Q3 | | 0.38 (0.29) | 0.18 |  |  | 0.42 (0.24) | 0.08 |  |  | 0.40 (0.24) | 0.09 |  |  |
| Q4 | | 0.22 (0.29) | 0.44 |  |  | 0.43 (0.24) | 0.07 |  |  | 0.41 (0.24) | 0.09 |  |  |
| Q5 | | -0.80 (0.29) | 0.005* |  |  | 0.15 (0.24) | 0.52 |  |  | 0.12 (0.24) | 0.63 |  |  |
| **Supplementary Table 10** (*continued*) | | |  |  |  |  |  |  |  |  |  |  |  |
| **KEC_ht_,** *^6^* **Nm/m^2^** | |  |  |  |  |  |  |  |  |  |  |  |  |
| *n* = 547 | Q2 | 1.50 (1.40) | 0.28 | 0.01 |  | 0.91 (1.12) | 0.42 | 0.39 |  | 0.92 (1.12) | 0.41 | 0.39 |  |
| Q3 | | 1.76 (1.43) | 0.22 |  |  | 1.07 (1.13) | 0.34 |  |  | 1.07 (1.13) | 0.34 |  |  |
| Q4 | | 1.81 (1.44) | 0.21 |  |  | 1.18 (1.14) | 0.30 |  |  | 1.19 (1.15) | 0.30 |  |  |
| Q5 | | -1.23 (1.45) | 0.40 |  |  | 1.76 (1.16) | 0.13 |  |  | 1.74 (1.17) | 0.14 |  |  |
| **KEI_ht_,** *^6^* **Nm/m^2^** | |  |  |  |  |  |  |  |  |  |  |  |  |
| *n* = 542 | Q2 | 2.21 (1.56) | 0.16 | 0.02 |  | 1.98 (1.25) | 0.11 | 0.39 |  | 2.19 (1.25) | 0.08 | 0.40 |  |
| Q3 | | 2.53 (1.59) | 0.11 |  |  | 2.02 (1.26) | 0.11 |  |  | 2.22 (1.26) | 0.08 |  |  |
| Q4 | | 2.44 (1.60) | 0.13 |  |  | 2.07 (1.28) | 0.11 |  |  | 2.50 (1.28) | 0.052 |  |  |
| Q5 | | -2.01 (1.62) | 0.22 |  |  | 2.00 (1.31) | 0.13 |  |  | 2.43 (1.31) | 0.07 |  |  |

*^1^* Data are presented as regression coefficients ($\beta$) $\pm$ standard error (SE), with Q1 of serum folate as the reference category. * denotes statistical significance. ALM: appendicular lean mass; ALM_ht_: height-adjusted ALM; FFM%: fat-free mass as a percentage of body weight; HGS_ht_: height-adjusted hand grip strength; KEC_ht_: height-adjusted knee extension concentric strength; KEI_ht_: height-adjusted knee extension isometric strength; SPPB: short physical performance battery.

*^2^* Model 1 is unadjusted.

*^3^* Model 2 for is adjusted for age (years), smoking status (never, former or current), race (White American, Black American, Other), physical activity level (not active, moderately active, active, highly active) and estimated glomerular filtration rate (ml/min/1.73m^2^) for all outcomes.

*^4^* Model 3 is additionally adjusted for use of folate supplements (none, <400μg/day, ≥400μg/day, unknown).

*^5^* ALM models are additionally adjusted for height (cm) and appendicular fat mass (kg).

*^6^* ALM_ht_, FFM%, extended SPPB score, HGS_ht_, KEC_ht_ and KEI_ht_ models are additionally adjusted for body mass index (kg/m^2^).

*^7^* Extended short physical performance battery (SPPB) score was transformed using a Box-Cox transformation in all models.

**Supplementary Table 11** Associations between serum folate and sarcopenic indices in men.^1^

|  | | **Men** | | | | | | | | | | | |
| --- | --- | --- | --- | --- | --- | --- | --- | --- | --- | --- | --- | --- | --- |
|  | | **Model 1***^2^* | | |  | **Model 2***^3^* | | |  | **Model 3***^4^* | | |  |
|  | | $\boldsymbol{\beta}$ $\pm$**SE** | ***p*** | **R^2^** |  | $\boldsymbol{\beta}$ $\pm$**SE** | ***p*** | **R^2^** |  | $\boldsymbol{\beta}$ $\pm$**SE** | ***p*** | **R^2^** |  |
| **ALM,**^5^ **kg** | |  |  |  |  |  |  |  |  |  |  |  |  |
| *n* = 645 | Q2 | 0.29 (0.53) | 0.58 | 0.05 |  | 0.24 (0.35) | 0.50 | 0.60 |  | 0.18 (0.35) | 0.60 | 0.60 |  |
| Q3 | | -0.62 (0.52) | 0.23 |  |  | -0.19 (0.34) | 0.58 |  |  | -0.20 (0.34) | 0.56 |  |  |
| Q4 | | -1.49 (0.53) | 0.005* |  |  | -0.28 (0.35) | 0.42 |  |  | -0.32 (0.35) | 0.36 |  |  |
| Q5 | | -2.27 (0.52) | <0.001* |  |  | -0.39 (0.35) | 0.27 |  |  | -0.43 (0.35) | 0.22 |  |  |
|  | |  |  |  |  |  |  |  |  |  |  |  |  |
| **ALM_ht_,***^6^* **kg/m^2^** | |  |  |  |  |  |  |  |  |  |  |  |  |
| *n* = 644 | Q2 | -0.028 (0.14) | 0.84 | 0.04 |  | 0.073 (0.11) | 0.49 | 0.46 |  | 0.061 (0.11) | 0.56 | 0.46 |  |
| Q3 | | -0.21 (0.14) | 0.12 |  |  | 0.032 (0.10) | 0.76 |  |  | 0.031 (0.10) | 0.77 |  |  |
| Q4 | | -0.40 (0.14) | 0.004* |  |  | -0.028 (0.11) | 0.79 |  |  | -0.031 (0.11) | 0.78 |  |  |
| Q5 | | -0.61 (0.14) | <0.001* |  |  | -0.056 (0.11) | 0.60 |  |  | -0.062 (0.11) | 0.57 |  |  |
|  | |  |  |  |  |  |  |  |  |  |  |  |  |
| **FFM%***^6^* | |  |  |  |  |  |  |  |  |  |  |  |  |
| *n* = 644 | Q2 | 1.08 (1.02) | 0.29 | 0.01 |  | 0.17 (0.63) | 0.79 | 0.62 |  | 0.11 (0.64) | 0.87 | 0.62 |  |
| Q3 | | 2.55 (1.00) | 0.011* |  |  | 0.063 (0.63) | 0.92 |  |  | 0.051 (0.63) | 0.94 |  |  |
| Q4 | | 0.95 (1.01) | 0.35 |  |  | -0.78 (0.64) | 0.22 |  |  | -0.78 (0.65) | 0.23 |  |  |
| Q5 | | 1.23 (1.00) | 0.22 |  |  | -0.60 (0.64) | 0.35 |  |  | -0.64 (0.65) | 0.33 |  |  |
|  | |  |  |  |  |  |  |  |  |  |  |  |  |
| **Extended SPPB Score***^6,7^* | |  |  |  |  |  |  |  |  |  |  |  |  |
| *n* = 625 | Q2 | 0.16 (0.32) | 0.61 | 0.04 |  | 0.050 (0.22) | 0.82 | 0.55 |  | 0.049 (0.22) | 0.82 | 0.56 |  |
| Q3 | | 0.024 (0.32) | 0.94 |  |  | -0.096 (0.22) | 0.66 |  |  | -0.12 (0.22) | 0.57 |  |  |
| Q4 | | -0.50 (0.32) | 0.11 |  |  | -0.039 (0.22) | 0.86 |  |  | -0.12 (0.22) | 0.60 |  |  |
| Q5 | | -1.25 (0.32) | <0.001* |  |  | -0.24 (0.22) | 0.29 |  |  | -0.33 (0.23) | 0.15 |  |  |
|  | |  |  |  |  |  |  |  |  |  |  |  |  |
| **HGS_ht_,** *^6^* **kg/m^2^** | |  |  |  |  |  |  |  |  |  |  |  |  |
| *n* = 665 | Q2 | -0.26 (0.40) | 0.52 | 0.04 |  | -0.21 (0.31) | 0.49 | 0.43 |  | -0.21 (0.31) | 0.49 | 0.43 |  |
| Q3 | | -0.47 (0.39) | 0.23 |  |  | -0.17 (0.30) | 0.57 |  |  | -0.19 (0.30) | 0.54 |  |  |
| Q4 | | -0.89 (0.39) | 0.024* |  |  | -0.098 (0.31) | 0.75 |  |  | -0.13 (0.31) | 0.68 |  |  |
| Q5 | | -1.72 (0.39) | <0.001* |  |  | -0.27 (0.31) | 0.39 |  |  | -0.31 (0.31) | 0.32 |  |  |
| **Supplementary Table 11** (*continued*) | | |  |  |  |  |  |  |  |  |  |  |  |
| **KEC_ht_,** *^6^* **Nm/m^2^** | |  |  |  |  |  |  |  |  |  |  |  |  |
| *n* = 479 | Q2 | 3.30 (2.11) | 0.12 | 0.04 |  | 3.63 (1.67) | 0.03* | 0.42 |  | 3.42 (1.69) | 0.043* | 0.41 |  |
| Q3 | | -0.27 (2.06) | 0.90 |  |  | 1.53 (1.63) | 0.35 |  |  | 1.43 (1.64) | 0.38 |  |  |
| Q4 | | -1.29 (2.13) | 0.54 |  |  | 3.19 (1.70) | 0.06 |  |  | 3.03 (1.71) | 0.08 |  |  |
| Q5 | | -5.97 (2.13) | 0.005* |  |  | 1.87 (1.75) | 0.29 |  |  | 1.86 (1.77) | 0.29 |  |  |
| **KEI_ht_,** *^6^* **Nm/m^2^** | |  |  |  |  |  |  |  |  |  |  |  |  |
| *n* = 476 | Q2 | 3.79 (2.39) | 0.11 | 0.04 |  | 4.57 (1.89) | 0.016* | 0.42 |  | 4.16 (1.89) | 0.028* | 0.43 |  |
| Q3 | | -0.30 (2.32) | 0.90 |  |  | 2.32 (1.84) | 0.21 |  |  | 2.12 (1.83) | 0.25 |  |  |
| Q4 | | -2.23 (2.40) | 0.35 |  |  | 3.32 (1.91) | 0.08 |  |  | 3.56 (1.91) | 0.06 |  |  |
| Q5 | | -6.73 (2.40) | 0.005* |  |  | 2.63 (1.97) | 0.18 |  |  | 2.70 (1.97) | 0.17 |  |  |

*^1^* Data are presented as regression coefficients ($\beta$) $\pm$ standard error (SE), with Q1 of serum folate as the reference category. * denotes statistical significance. ALM: appendicular lean mass; ALM_ht_: height-adjusted ALM; FFM%: fat-free mass as a percentage of body weight; HGS_ht_: height-adjusted hand grip strength; KEC_ht_: height-adjusted knee extension concentric strength; KEI_ht_: height-adjusted knee extension isometric strength; SPPB: short physical performance battery.

*^2^* Model 1 is unadjusted.

*^3^* Model 2 for is adjusted for age (years), smoking status (never, former or current), race (White American, Black American, Other), physical activity level (not active, moderately active, active, highly active) and estimated glomerular filtration rate (ml/min/1.73m^2^) for all outcomes.

*^4^* Model 3 is additionally adjusted for use of folate supplements (none, <400μg/day, ≥400μg/day, unknown).

*^5^* ALM models are additionally adjusted for height (cm) and appendicular fat mass (kg).

*^6^* ALM_ht_, FFM%, extended SPPB score, HGS_ht_, KEC_ht_ and KEI_ht_ models are additionally adjusted for body mass index (kg/m^2^).

*^7^* Extended short physical performance battery (SPPB) score was transformed using a Box-Cox transformation in all models.

**Supplementary Table 12** Associations between nutritional biomarkers and muscle strength or function in women.*^1^*

|  | **Women** | | | | | | | | | | | | | | | | |  |
| --- | --- | --- | --- | --- | --- | --- | --- | --- | --- | --- | --- | --- | --- | --- | --- | --- | --- | --- |
|  |  | **Model 1***^3^* | | | |  | **Model 2***^4^* | | | |  | **Model 3***^5^* | | | | | |  |
| **Extended SPPB Score***^2^* | ***n*** | $\boldsymbol{\beta}$ $\pm$**SE** | | **R^2^** | ***p*** |  | $\boldsymbol{\beta}$ $\pm$**SE** | | **R^2^** | ***p*** |  | $\boldsymbol{\beta}$ $\pm$**SE** | | | | **R^2^** | ***p*** |  |
| Vitamin D, ng/mL | 667 | -0.0043 $\pm$0.0075 | | 0.0005 | 0.56 |  | -0.00063 $\pm$0.0048 | | 0.62 | 0.90 |  | -0.00091 $\pm$0.0048 | | | | 0.63 | 0.85 |  |
| Vitamin B12, pg/mL | 675 | -0.00041 $\pm$0.00031 | | 0.003 | 0.18 |  | -0.00010 $\pm$0.00019 | | 0.62 | 0.60 |  | -0.000085 $\pm$0.00019 | | | | 0.63 | 0.66 |  |
| Magnesium, mg/dL | 699 | 1.32 $\pm$0.50 | | 0.01 | 0.009* |  | 1.07 $\pm$0.32 | | 0.62 | 0.001* |  | 0.85 $\pm$0.32 | | | | 0.63 | 0.009* |  |
| Potassium, mmol/L | 710 | -1.15 $\pm$0.34 | | 0.02 | 0.001* |  | -0.32 $\pm$0.21 | | 0.63 | 0.13 |  | -0.26 $\pm$0.21 | | | | 0.64 | 0.23 |  |
| Calcium, mg/dL | 710 | -1.07 $\pm$0.22 | | 0.03 | <0.001* |  | -0.40 $\pm$0.14 | | 0.63 | 0.005* |  | -0.28 $\pm$0.14 | | | | 0.64 | 0.048* |  |
| Iron, $\mu$g/dL | 699 | 0.0064 $\pm$0.0035 | | 0.005 | 0.07 |  | 0.0035 $\pm$0.0023 | | 0.62 | 0.12 |  | 0.0030 $\pm$0.0022 | | | | 0.63 | 0.17 |  |
|  |  |  |  |  |  |  |  |  |  |  |  |  | | |  |  |  |  |
| **HGS_ht_, kg/m^2^** |  |  |  |  |  |  |  |  |  |  |  |  | | |  |  |  |  |
| Vitamin D, ng/mL | 668 | -0.010 $\pm$0.007 | | 0.003 | 0.13 |  | 0.0049 $\pm$0.0057 | | 0.35 | 0.39 |  | 0.0046 $\pm$0.0057 | | | | 0.34 | 0.41 |  |
| Vitamin B12, pg/mL | 698 | -0.000069 $\pm$0.00027 | | 0.0001 | 0.80 |  | 0.00022 $\pm$0.00023 | | 0.35 | 0.33 |  | 0.00023 $\pm$0.00023 | | | | 0.35 | 0.31 |  |
| Magnesium, mg/dL | 723 | -0.47 $\pm$0.45 | | 0.002 | 0.30 |  | -0.00037 $\pm$0.37 | | 0.36 | 1.00 |  | -0.11 $\pm$0.38 | | | | 0.36 | 0.76 |  |
| Potassium, mmol/L | 787 | -0.62 $\pm$0.27 | | 0.007 | 0.023* |  | 0.012 $\pm$0.22 | | 0.37 | 0.96 |  | 0.065 $\pm$0.23 | | | | 0.37 | 0.78 |  |
| Calcium, mg/dL | 787 | -0.28 $\pm$0.19 | | 0.003 | 0.14 |  | 0.014 $\pm$0.16 | | 0.37 | 0.93 |  | 0.056 $\pm$0.16 | | | | 0.37 | 0.73 |  |
| Iron, $\mu$g/dL | 723 | -0.0036 $\pm$0.0031 | | 0.002 | 0.25 |  | 0.0011 $\pm$0.0026 | | 0.36 | 0.66 |  | 0.00090 $\pm$0.0026 | | | | 0.36 | 0.73 |  |
|  |  |  |  |  |  |  |  |  |  |  |  |  | |  | |  |  |  |
| **KEC_ht_, Nm/m^2^** | |  |  |  |  |  |  |  |  |  |  |  | |  | |  |  |  |
| Vitamin D, ng/mL | 545 | -0.0025 $\pm$0.033 | | <0.0001 | 0.94 |  | 0.052 $\pm$0.026 | | 0.39 | 0.046* |  | 0.048 $\pm$0.026 | | | | 0.39 | 0.07 |  |
| Vitamin B12, pg/mL | 533 | -0.00099 $\pm$0.0013 | | 0.001 | 0.45 |  | 0.00063 $\pm$0.0011 | | 0.38 | 0.55 |  | 0.00059 $\pm$0.0011 | | | | 0.38 | 0.59 |  |
| Magnesium, mg/dL | 552 | -5.62 $\pm$2.23 | | 0.01 | 0.012* |  | -2.98 $\pm$1.80 | | 0.39 | 0.10 |  | -3.31 $\pm$1.83 | | | | 0.39 | 0.07 |  |
| Potassium, mmol/L | 552 | 0.85 $\pm$1.64 | | 0.0005 | 0.60 |  | 2.07 $\pm$1.31 | | 0.39 | 0.12 |  | 2.07 $\pm$1.31 | | | | 0.39 | 0.11 |  |
| Calcium, mg/dL | 552 | -0.49 $\pm$1.11 | | 0.0004 | 0.66 |  | 1.11 $\pm$0.89 | | 0.39 | 0.21 |  | 1.14 $\pm$0.91 | | | | 0.39 | 0.21 |  |
| Iron, $\mu$g/dL | 552 | -0.0068 $\pm$0.016 | | 0.0003 | 0.67 |  | 0.0083 $\pm$0.013 | | 0.39 | 0.52 |  | 0.0078 $\pm$0.013 | | | | 0.39 | 0.54 |  |
| **Supplementary Table 12** (*continued*) | | | | | | | | | | | | | | | | | | |
| **KEI_ht_, Nm/m^2^** | |  |  |  |  |  |  |  |  |  |  |  |  | | |  |  | |
| Vitamin D, ng/mL | 540 | -0.045 $\pm$0.036 | | 0.003 | 0.22 |  | 0.023 $\pm$0.029 | | 0.39 | 0.44 |  | 0.021 $\pm$0.029 | | | | 0.40 | 0.48 |  |
| Vitamin B12, pg/mL | 528 | -0.00081 $\pm$0.0015 | | 0.0006 | 0.58 |  | 0.0011 $\pm$0.0012 | | 0.39 | 0.34 |  | 0.0011 $\pm$0.0012 | | | | 0.40 | 0.34 |  |
| Magnesium, mg/dL | 547 | -7.87 $\pm$2.50 | | 0.02 | 0.002* |  | -4.31 $\pm$2.02 | | 0.39 | 0.033* |  | -3.38 $\pm$2.05 | | | | 0.40 | 0.10 |  |
| Potassium, mmol/L | 547 | 1.45 $\pm$1.84 | | 0.001 | 0.43 |  | 3.10 $\pm$1.46 | | 0.39 | 0.034* |  | 3.00 $\pm$1.46 | | | | 0.40 | 0.041* |  |
| Calcium, mg/dL | 547 | 0.056 $\pm$1.25 | | <0.0001 | 0.96 |  | 1.58 $\pm$1.00 | | 0.39 | 0.11 |  | 1.25 $\pm$1.01 | | | | 0.39 | 0.22 |  |
| Iron, $\mu$g/dL | 547 | -0.035 $\pm$0.018 | | 0.007 | 0.047* |  | -0.012 $\pm$0.014 | | 0.39 | 0.41 |  | -0.010 $\pm$0.015 | | | | 0.39 | 0.48 |  |

*^1^* Data are presented as regression coefficients ($\beta$) $\pm$ standard error (SE). * denotes statistical significance. HGS_ht_: height-adjusted hand grip strength; KEC_ht_: height-adjusted knee extension concentric strength; KEI_ht_: height-adjusted knee extension isometric strength.
*^2^* Extended short physical performance battery (SPPB) score was transformed using a Box-Cox transformation in all models.

*^3^* Model 1 is unadjusted.

*^4^* Model 2 is adjusted for age (years), body mass index (kg/m^2^) smoking status (never, former or current), race (White American, Black American, Other), physical activity level (not active, moderately active, active, highly active) and estimated glomerular filtration rate (ml/min/1.73m^2^) for all outcomes.

*^5^* Model 3 is additionally adjusted for use of dietary supplements for the corresponding nutrient.

**Supplementary Table 13** Associations between nutritional biomarkers and muscle strength or function in Men.^1^

|  | **Men** | | | | | | | | | | | | | | | |
| --- | --- | --- | --- | --- | --- | --- | --- | --- | --- | --- | --- | --- | --- | --- | --- | --- |
|  |  | **Model 1***^3^* | | | |  | **Model 2***^4^* | | | |  | **Model 3***^5^* | | | | |
| **Extended SPPB Score***^2^* | ***n*** | $\boldsymbol{\beta}$ $\pm$**SE** | | **R^2^** | ***p*** |  | $\boldsymbol{\beta}$ $\pm$**SE** | | **R^2^** | ***p*** |  | $\boldsymbol{\beta}$ $\pm$**SE** | **R^2^** | | ***p*** | |
| Vitamin D, ng/mL | 601 | -0.024 $\pm$0.008 | | 0.01 | 0.004* |  | -0.0037 $\pm$0.0060 | | 0.54 | 0.54 |  | -0.0055 $\pm$0.0062 | 0.54 | | 0.37 | |
| Vitamin B12, pg/mL | 626 | -0.0010 $\pm$0.0003 | | 0.02 | 0.002* |  | -0.00042 $\pm$0.00023 | | 0.56 | 0.06 |  | -0.00045 $\pm$0.00023 | 0.57 | | 0.05 | |
| Magnesium, mg/dL | 635 | 1.36 $\pm$0.47 | | 0.01 | 0.004* |  | 0.92 $\pm$0.32 | | 0.56 | 0.004* |  | 0.85 $\pm$0.32 | 0.56 | | 0.009* | |
| Potassium, mmol/L | 641 | -0.42 $\pm$0.32 | | 0.003 | 0.19 |  | -0.21 $\pm$0.21 | | 0.56 | 0.32 |  | -0.19 $\pm$0.21 | 0.56 | | 0.37 | |
| Calcium, mg/dL | 641 | -0.36 $\pm$0.23 | | 0.004 | 0.12 |  | -0.77 $\pm$0.15 | | 0.57 | <0.001* |  | -0.73 $\pm$0.16 | 0.58 | | <0.001* | |
| Iron, $\mu$g/dL | 635 | 0.012 $\pm$0.003 | | 0.02 | <0.001* |  | 0.0041 $\pm$0.0022 | | 0.56 | 0.06 |  | 0.0043 $\pm$0.0022 | 0.56 | | 0.045* | |
|  |  |  |  |  |  |  |  |  |  |  |  |  | |  |  |  |
| **HGS_ht_, kg/m^2^** |  |  |  |  |  |  |  |  |  |  |  |  | |  |  |  |
| Vitamin D, ng/mL | 604 | -0.045 $\pm$0.011 | | 0.03 | < 0.001* |  | -0.011 $\pm$0.008 | | 0.43 | 0.20 |  | -0.011 $\pm$0.009 | 0.43 | | 0.21 | |
| Vitamin B12, pg/mL | 666 | -0.00088 $\pm$0.00041 | | 0.007 | 0.034* |  | -0.000085 $\pm$0.00032 | | 0.44 | 0.79 |  | -0.00011 $\pm$0.00033 | 0.44 | | 0.74 | |
| Magnesium, mg/dL | 677 | 0.50 $\pm$0.57 | | 0.001 | 0.38 |  | 0.64 $\pm$0.43 | | 0.44 | 0.14 |  | 0.64 $\pm$0.44 | 0.44 | | 0.14 | |
| Potassium, mmol/L | 736 | -0.45 $\pm$0.36 | | 0.002 | 0.22 |  | 0.13 $\pm$0.27 | | 0.45 | 0.64 |  | 0.15 $\pm$0.28 | 0.45 | | 0.59 | |
| Calcium, mg/dL | 736 | 0.11 $\pm$0.26 | | 0.0002 | 0.68 |  | -0.20 $\pm$0.19 | | 0.45 | 0.31 |  | -0.19 $\pm$0.20 | 0.45 | | 0.32 | |
| Iron, $\mu$g/dL | 677 | 0.011 $\pm$0.004 | | 0.01 | 0.004* |  | 0.0041 $\pm$0.0029 | | 0.44 | 0.17 |  | 0.0040 $\pm$0.0030 | 0.43 | | 0.18 | |
|  |  |  |  |  |  |  |  |  |  |  |  |  | |  |  |  |
| **KEC_ht_, Nm/m^2^** | |  |  |  |  |  |  |  |  |  |  |  | |  |  |  |
| Vitamin D, ng/mL | 479 | -0.15 $\pm$0.06 | | 0.02 | 0.007* |  | -0.0068 $\pm$0.045 | | 0.41 | 0.88 |  | 0.0097 $\pm$0.046 | 0.42 | | 0.83 | |
| Vitamin B12, pg/mL | 479 | -0.0067 $\pm$0.0022 | | 0.02 | 0.002* |  | -0.0019 $\pm$0.0017 | | 0.42 | 0.28 |  | -0.0018 $\pm$0.0018 | 0.42 | | 0.30 | |
| Magnesium, mg/dL | 488 | -1.32 $\pm$3.06 | | 0.0004 | 0.67 |  | -2.12 $\pm$2.38 | | 0.42 | 0.37 |  | -1.17 $\pm$2.42 | 0.42 | | 0.63 | |
| Potassium, mmol/L | 490 | 2.90 $\pm$2.25 | | 0.003 | 0.20 |  | 5.03 $\pm$1.77 | | 0.42 | 0.005* |  | 4.85 $\pm$1.76 | 0.43 | | 0.006* | |
| Calcium, mg/dL | 490 | 0.71 $\pm$1.69 | | 0.0004 | 0.68 |  | -0.88 $\pm$1.31 | | 0.41 | 0.50 |  | -0.72 $\pm$1.34 | 0.42 | | 0.59 | |
| Iron, $\mu$g/dL | 488 | 0.027 $\pm$0.021 | | 0.003 | 0.20 |  | 0.010 $\pm$0.016 | | 0.42 | 0.53 |  | 0.0068 $\pm$0.016 | 0.42 | | 0.68 | |
| **Supplementary Table 13** (*continued*) | | | | | | | | | | | | | | | | |
| **KEI_ht_, Nm/m^2^** | |  |  |  |  |  |  |  |  |  |  |  | |  |  |  |
| Vitamin D, ng/mL | 476 | -0.24 $\pm$0.06 | | 0.03 | <0.001* |  | -0.064 $\pm$0.052 | | 0.41 | 0.21 |  | -0.048 $\pm$0.051 | 0.43 | | 0.35 | |
| Vitamin B12, pg/mL | 476 | -0.0071 $\pm$0.0025 | | 0.02 | 0.004* |  | -0.0024 $\pm$0.0020 | | 0.42 | 0.22 |  | -0.0024 $\pm$0.0020 | 0.43 | | 0.23 | |
| Magnesium, mg/dL | 485 | -4.81 $\pm$3.47 | | 0.004 | 0.17 |  | -4.77 $\pm$2.70 | | 0.42 | 0.08 |  | -3.31 $\pm$2.73 | 0.43 | | 0.23 | |
| Potassium, mmol/L | 487 | 3.91 $\pm$2.56 | | 0.005 | 0.13 |  | 6.35 $\pm$2.00 | | 0.43 | 0.002* |  | 6.08 $\pm$1.99 | 0.43 | | 0.002* | |
| Calcium, mg/dL | 487 | 1.71 $\pm$1.92 | | 0.002 | 0.37 |  | -0.30 $\pm$1.49 | | 0.41 | 0.84 |  | -0.74 $\pm$1.50 | 0.43 | | 0.62 | |
| Iron, $\mu$g/dL | 485 | 0.011 $\pm$0.024 | | 0.0005 | 0.63 |  | -0.00019 $\pm$0.019 | | 0.42 | 0.99 |  | -0.0066 $\pm$0.018 | 0.43 | | 0.72 | |

*^1^* Data are presented as regression coefficients ($\beta$) $\pm$ standard error (SE). * denotes statistical significance. HGS_ht_: height-adjusted hand grip strength; KEC_ht_: height-adjusted knee extension concentric strength; KEI_ht_: height-adjusted knee extension isometric strength.
*^2^* Extended short physical performance battery (SPPB) score was transformed using a Box-Cox transformation in all models.

*^3^* Model 1 is unadjusted.

*^4^* Model 2 is adjusted for age (years), body mass index (kg/m^2^) smoking status (never, former or current), race (White American, Black American, Other), physical activity level (not active, moderately active, active, highly active) and estimated glomerular filtration rate (ml/min/1.73m^2^) for all outcomes.

*^5^* Model 3 is additionally adjusted for use of dietary supplements for the corresponding nutrient.

**Supplementary Table 14** Biomarkers found to be significantly associated with muscle outcomes in all women (left), and results for associations in older women only (≥ 65 years; right).^1^

|  |  | **All Women** | | |  |  | **Women ≥ 65 years** | | |
| --- | --- | --- | --- | --- | --- | --- | --- | --- | --- |
| **Continuous Models** | ***n*** | $\boldsymbol{\beta\pm}$**SE** | ***p*** | **R^2^** |  | ***n*** | $\boldsymbol{\beta}$ $\boldsymbol{\pm}$ **SE** | ***p*** | **R^2^** |
| **Haemoglobin** |  |  |  |  |  |  |  |  |  |
| ALM_ht_ | 704 | -0.057 $\pm$0.027 | 0.034* | 0.42 |  | 472 | -0.057 (0.031) | 0.065 | 0.37 |
| _ext_SPPB | 704 | 0.15 $\pm$0.06 | 0.019* | 0.63 |  | 481 | 0.18 (0.07) | 0.014* | 0.55 |
|  |  |  |  |  |  |  |  |  |  |
| **Ferritin** |  |  |  |  |  |  |  |  |  |
| KEC_ht_ | 551 | 0.012 $\pm$0.005 | 0.031* | 0.40 |  | 373 | 0.0068 (0.0058) | 0.24 | 0.23 |
| KEI_ht_ | 546 | 0.013 $\pm$0.006 | 0.031* | 0.40 |  | 368 | 0.0059 (0.0065) | 0.37 | 0.24 |
|  |  |  |  |  |  |  |  |  |  |
| **Albumin** |  |  |  |  |  |  |  |  |  |
| ALM | 709 | -0.64 $\pm$ 0.23 | 0.005* | 0.54 |  | 477 | -0.93 (0.27) | 0.001* | 0.50 |
| ALM_ht_ | 709 | -0.26 $\pm$0.08 | 0.001* | 0.43 |  | 477 | -0.38 (0.10) | <0.001* | 0.39 |
| extSPPB | 710 | -0.50 $\pm$0.19 | 0.008* | 0.63 |  | 487 | -0.41 (0.24) | 0.08 | 0.55 |
| KEI_ht_ | 547 | 2.89 $\pm$1.34 | 0.032* | 0.39 |  | 369 | 0.91 (1.61) | 0.57 | 0.24 |
|  |  |  |  |  |  |  |  |  |  |
| **Creatinine (All)** |  |  |  |  |  |  |  |  |  |
| ALM | 709 | 0.78 $\pm$ 0.39 | 0.045* | 0.54 |  | 477 | 0.63 (0.40) | 0.12 | 0.48 |
| FFM% | 709 | 1.79 $\pm$0.89 | 0.044* | 0.71 |  | 477 | 1.06 (0.98) | 0.28 | 0.70 |
|  |  |  |  |  |  |  |  |  |  |
| **Creatinine (eGFR ≥ 60)** |  |  |  |  |  |  |  |  |  |
| ALM | 615 | 3.26 (0.76) | <0.001* | 0.55 |  | 390 | 2.44 (0.87) | 0.005* | 0.50 |
| ALM_ht_ | 615 | 1.07 (0.27) | <0.001* | 0.43 |  | 390 | 0.81 (0.32) | 0.011* | 0.37 |
| FFM% | 615 | 5.41 (1.74) | 0.002* | 0.72 |  | 390 | 4.84 (2.11) | 0.022* | 0.71 |
|  |  |  |  |  |  |  |  |  |  |
| **B12** |  |  |  |  |  |  |  |  |  |
| ALM_ht_ | 686 | 0.00017 $\pm$0.00008 | 0.035* | 0.43 |  | 463 | 0.00021 (0.00009) | 0.025* | 0.38 |
|  |  |  |  |  |  |  |  |  |  |
| **Magnesium** |  |  |  |  |  |  |  |  |  |
| _ext_SPPB | 699 | 0.85 $\pm$0.32 | 0.009* | 0.63 |  | 476 | 0.97 (0.39) | 0.013* | 0.54 |
|  |  |  |  |  |  |  |  |  |  |
| **Potassium** |  |  |  |  |  |  |  |  |  |
| KEI_ht_ | 547 | 3.00 $\pm$1.46 | 0.041* | 0.40 |  | 369 | 2.60 (1.56) | 0.10 | 0.25 |
|  |  |  |  |  |  |  |  |  |  |
| **Calcium** |  |  |  |  |  |  |  |  |  |
| ALM | 709 | -0.37 $\pm$0.17 | 0.031* | 0.54 |  | 477 | -0.43 (0.20) | 0.03* | 0.49 |
| ALM_ht_ | 709 | -0.16 $\pm$0.06 | 0.008* | 0.43 |  | 477 | -0.18 (0.07) | 0.013* | 0.38 |
| _ext_SPPB | 710 | -0.28 $\pm$0.14 | 0.048* | 0.64 |  | 487 | -0.30 (0.17) | 0.08 | 0.56 |
|  |  |  |  |  |  |  |  |  |  |
| **Categorical Models** |  |  |  |  |  |  |  |  |  |
| **Folate, ng/mL (vs. Q1)** |  |  |  |  |  |  |  |  |  |
| FFM% | 703 |  |  |  |  | 472 |  |  |  |
| Q2 |  | 1.24 (0.55) | 0.025* | 0.71 |  |  | 1.72 (0.69) | 0.014* | 0.70 |
| Q3 |  | 0.80 (0.55) | 0.15 |  |  |  | 1.08 (0.69) | 0.12 |  |
| Q4 |  | 1.32 (0.56) | 0.019* |  |  |  | 1.68 (0.68) | 0.014* |  |
| Q5 |  | 0.72 (0.57) | 0.21 |  |  |  | 0.73 (0.65) | 0.27 |  |
| **Supplementary Table 14** (*continued)* | | |  |  |  |  |  |  |  |
| _ext_SPPB | 691 |  |  |  |  | 469 |  |  |  |
| Q2 |  | 0.41 (0.20) | 0.038* | 0.63 |  |  | 0.57 (0.26) | 0.027* | 0.55 |
| Q3 |  | 0.36 (0.20) | 0.07 |  |  |  | 0.39 (0.25) | 0.12 |  |
| Q4 |  | 0.19 (0.20) | 0.35 |  |  |  | 0.18 (0.25) | 0.47 |  |
| Q5 |  | -0.17 (0.20) | 0.40 |  |  |  | -0.0089 (0.24) | 0.97 |  |
| HGS_ht_ | 715 |  |  |  |  | 480 |  |  |  |
| Q2 |  | 0.56 (0.24) | 0.018* | 0.36 |  |  | 0.26 (0.30) | 0.38 | 0.26 |
| Q3 |  | 0.40 (0.24) | 0.09 |  |  |  | 0.40 (0.30) | 0.18 |  |
| Q4 |  | 0.41 (0.24) | 0.09 |  |  |  | 0.24 (0.29) | 0.42 |  |
| Q5 |  | 0.12 (0.24) | 0.63 |  |  |  | 0.011 (0.28) | 0.97 |  |

*^1^* Data are presented as regression coefficients ($\beta$) $\pm$ standard error (SE) from fully adjusted regression models as described in Supplementary Tables 4 – 13. All biomarkers, except folate, were entered into regression models as continuous variables. Folate was categorised into quintiles, with Q1 of serum folate set as the reference category. * denotes statistical significance. ALM: appendicular lean mass; ALM_ht_: height-adjusted ALM; FFM%: fat-free mass as a percentage of body weight; HGS_ht_: height-adjusted hand grip strength; KEC_ht_: height-adjusted knee extension concentric strength; KEI_ht_: height-adjusted knee extension isometric strength; SPPB: short physical performance battery.

**Supplementary Table 15** Biomarkers found to be significantly associated with muscle outcomes in all men (left), and results for associations in older men only (≥ 65 years; right).^1^

|  | |  | | **All Men** | | | | | |  | |  | | **Men ≥ 65 years** | | | | | |
| --- | --- | --- | --- | --- | --- | --- | --- | --- | --- | --- | --- | --- | --- | --- | --- | --- | --- | --- | --- |
| **Continuous Models** | | ***n*** | | $\boldsymbol{\beta\pm}$**SE** | | ***p*** | | **R^2^** | |  | | ***n*** | | $\boldsymbol{\beta}$ $\boldsymbol{\pm}$ **SE** | ***p*** | | **R^2^** | | |
| **Haemoglobin** | |  | |  | |  | |  | |  | |  | |  |  | |  | | |
| ALM | | 651 | | 0.20 $\pm$0.09 | | 0.021* | | 0.60 | |  | | 465 | | 0.24 (0.09) | 0.011* | | 0.49 | | |
| _ext_SPPB | | 633 | | 0.13 $\pm$0.06 | | 0.024* | | 0.56 | |  | | 448 | | 0.16 (0.06) | 0.013* | | 0.45 | | |
| HGS_ht_ | | 723 | | 0.25 $\pm$0.07 | | 0.001* | | 0.46 | |  | | 514 | | 0.26 (0.08) | 0.002* | | 0.31 | | |
|  |  | |  | |  | |  | |  | |  | |  | | |  | |  |  |
| **Albumin** | |  | |  | |  | |  | |  | |  | |  |  | |  | | |
| ALM | | 658 | | -0.94 $\pm$0.32 | | 0.004* | | 0.60 | |  | | 470 | | -0.90 (0.36) | 0.013* | | 0.49 | | |
| ALM_ht_ | | 657 | | -0.24 $\pm$0.10 | | 0.017* | | 0.47 | |  | | 470 | | -0.29 (0.12) | 0.015* | | 0.35 | | |
| _ext_SPPB | | 641 | | -0.60 $\pm$0.20 | | 0.003* | | 0.56 | |  | | 453 | | -0.62 (0.25) | 0.013* | | 0.45 | | |
|  |  | |  | |  | |  | |  | |  | |  | | |  | |  |  |
| **Creatinine (All)** | |  | |  | |  | |  | |  | |  | |  |  | |  | | |
| ALM | | 658 | | 1.28 $\pm$0.41 | | 0.002* | | 0.59 | |  | | 470 | | 1.08 (0.40) | 0.008* | | 0.48 | | |
| ALM_ht_ | | 657 | | 0.34 $\pm$0.12 | | 0.006* | | 0.46 | |  | | 470 | | 0.28 (0.13) | 0.034* | | 0.34 | | |
| FFM% | | 657 | | 2.64 $\pm$0.75 | | <0.001* | | 0.62 | |  | | 470 | | 2.51 (0.80) | 0.002* | | 0.58 | | |
|  |  | |  | |  | |  | |  | |  | |  | | |  | |  |  |
| **Creatinine (eGFR ≥ 60)** | |  | |  | |  | |  | |  | |  | |  |  | |  | | |
| ALM | | 543 | | 3.27 (0.87) | | <0.001* | | 0.59 | |  | | 357 | | 2.27 (0.97) | 0.02* | | 0.45 | | |
| ALM_ht_ | | 542 | | 0.97 (0.26) | | <0.001* | | 0.47 | |  | | 357 | | 0.70 (0.31) | 0.024* | | 0.35 | | |
| _ext_SPPB | | 537 | | 1.66 (0.54) | | 0.002* | | 0.54 | |  | | 351 | | 1.80 (0.68) | 0.008* | | 0.43 | | |
| HGS_ht_ | | 606 | | 1.55 (0.74) | | 0.037* | | 0.44 | |  | | 396 | | 1.50 (0.90) | 0.10 | | 0.27 | | |
| KEC_ht_ | | 421 | | 9.86 (4.30) | | 0.022* | | 0.41 | |  | | 262 | | 13.04 (4.60) | 0.005* | | 0.22 | | |
| KEI_ht_ | | 421 | | 12.68 (4.85) | | 0.009* | | 0.43 | |  | | 262 | | 15.76 (5.24) | 0.003* | | 0.28 | | |
|  |  | |  | |  | |  | |  | |  | |  | | |  | |  |  |
| **Magnesium** | |  | |  | |  | |  | |  | |  | |  |  | |  | | |
| _ext_SPPB | | 635 | | 0.85 $\pm$0.32 | | 0.009* | | 0.56 | |  | | 447 | | 0.68 (0.39) | 0.08 | | 0.45 | | |
|  |  | |  | |  | |  | |  | |  | |  | | |  | |  |  |
| **Potassium** | |  | |  | |  | |  | |  | |  | |  |  | |  | | |
| KEC_ht_ | | 490 | | 4.85 $\pm$1.76 | | 0.006* | | 0.43 | |  | | 331 | | 3.66 (1.81) | 0.044* | | 0.23 | | |
| KEI_ht_ | | 487 | | 6.08 $\pm$1.99 | | 0.002* | | 0.43 | |  | | 328 | | 5.26 (2.09) | 0.012* | | 0.26 | | |
|  |  | |  | |  | |  | |  | |  | |  | | |  | |  |  |
| **Calcium** | |  | |  | |  | |  | |  | |  | |  |  | |  | | |
| ALM | | 658 | | -0.60 $\pm$0.23 | | 0.011* | | 0.60 | |  | | 470 | | -0.68 (0.27) | 0.013* | | 0.49 | | |
| ALM_ht_ | | 657 | | -0.20 $\pm$0.07 | | 0.006* | | 0.47 | |  | | 470 | | -0.25 (0.09) | 0.005* | | 0.36 | | |
| _ext_SPPB | | 641 | | -0.73 $\pm$0.16 | | <0.001* | | 0.58 | |  | | 453 | | -0.60 (0.20) | 0.002* | | 0.46 | | |
|  |  | |  | |  | |  | |  | |  | |  | | |  | |  |  |
| **Iron** | |  | |  | |  | |  | |  | |  | |  |  | |  | | |
| ALM_ht_ | | 655 | | 0.0021 $\pm$0.0010 | | 0.038* | | 0.46 | |  | | 468 | | 0.0034 (0.0012) | 0.005* | | 0.36 | | |
| _ext_SPPB | | 635 | | 0.0043 $\pm$0.0022 | | 0.045* | | 0.56 | |  | | 447 | | 0.0041 (0.0027) | 0.12 | | 0.44 | | |
|  |  | |  | |  | |  | |  | |  | |  | | |  | |  |  |
| **Categorical Models** | |  | |  | |  | |  | |  | |  | |  |  | |  | | |
| **Hba1c (>6% vs. ≤6%)** | |  | |  | |  | |  | |  | |  | |  |  | |  | | |
| ALM_ht_ | | 593 | | -0.21 (0.09) | | 0.023* | | 0.46 | |  | | 421 | | -0.22 (0.10) | 0.033* | | 0.35 | | |
| **Supplementary Table 15** *(continued)* | | | | | | | | | |  | |  | |  | | | | | |
|  |  | |  | |  | |  | |  | |  | |  | | |  | |  |  |
| _ext_SPPB | | 605 | | -0.40 (0.18) | | 0.027* | | 0.55 | |  | | 422 | | -0.48 (0.21) | 0.019* | | 0.44 | | |
| HGS_ht_ | | 611 | | -0.56 (0.26) | | 0.031* | | 0.44 | |  | | 427 | | -0.60 (0.28) | 0.035* | | 0.28 | | |
|  |  | |  | |  | |  | |  | |  | |  | | |  | |  |  |
| **Folate, ng/mL (vs. Q1)** | |  | |  | |  | |  | |  | |  | |  |  | |  | | |
| KEC_ht_ | | 479 | |  | |  | |  | |  | | 320 | |  |  | |  | | |
| Q2 | |  | | 3.42 (1.69) | | 0.043* | | 0.41 | |  | |  | | 4.27 (1.96) | 0.03* | | 0.23 | | |
| Q3 | |  | | 1.43 (1.64) | | 0.38 | |  | |  | |  | | 1.88 (1.83) | 0.31 | |  | | |
| Q4 | |  | | 3.03 (1.71) | | 0.08 | |  | |  | |  | | 3.81 (1.81) | 0.036* | |  | | |
| Q5 | |  | | 1.86 (1.77) | | 0.29 | |  | |  | |  | | 2.50 (1.78) | 0.16 | |  | | |
| KEI_ht_ | | 476 | |  | |  | |  | |  | | 317 | |  |  | |  | | |
| Q2 | |  | | 4.16 (1.89) | | 0.028* | | 0.43 | |  | |  | | 5.58 (2.24) | 0.013* | | 0.27 | | |
| Q3 | |  | | 2.12 (1.83) | | 0.25 | |  | |  | |  | | 3.05 (2.07) | 0.14 | |  | | |
| Q4 | |  | | 3.56 (1.91) | | 0.06 | |  | |  | |  | | 4.78 (2.06) | 0.021* | |  | | |
| Q5 | |  | | 2.70 (1.97) | | 0.17 | |  | |  | |  | | 3.78 (2.02) | 0.06 | |  | | |

*^1^* Data are presented as regression coefficients ($\beta$) $\pm$ standard error (SE) from fully adjusted regression models as described in Table 2 and Supplementary Tables 4 – 13. All biomarkers, except HbA1c and folate, were entered into regression models as continuous variables. HbA1c was entered as a categorical variable (≤6%, >6%), with ≤6% set as the reference category. Folate was categorised into quintiles, with Q1 of serum folate set as the reference category. * denotes statistical significance. ALM: appendicular lean mass; ALM_ht_: height-adjusted ALM; FFM%: fat-free mass as a percentage of body weight; HGS_ht_: height-adjusted hand grip strength; KEC_ht_: height-adjusted knee extension concentric strength; KEI_ht_: height-adjusted knee extension isometric strength; SPPB: short physical performance battery.

**Supplementary Figure 1** Correlation matrices showing Spearman’s rho correlation coefficients between measures of lean mass, muscle strength and muscle function in women (**A**) and men (**B**). Positive correlations are shown in blue, negative corelations are shown in red, with darker colours indicating a stronger correlation. ALM: appendicular lean mass; ALM_ht_: height-adjusted ALM; _ext_SPPB: extended short physical performance battery; FFM%: fat-free mass as a percentage of body weight; HGS_ht_: height-adjusted hand grip strength; KEC_ht_: height-adjusted knee extension concentric strength; KEI_ht_: height-adjusted knee extension isometric strength. ^*^ p < 0.5, ^**^ p < 0.01, ^***^ p < 0.001.

**Supplementary Figure 2** Associations between serum creatinine and muscle outcomes for all participants (black), and from sensitivity analysis including participants with an estimated glomerular filtration rate (eGFR) $\geq$ 60 mL/min/1.73m^2^ (grey) in women (left) and men (right). Forest plots show standardised $\beta$ coefficient (95% confidence intervals). Extended short physical performance battery score (_ext_SPPB) was transformed using a Box-Cox transformation before standardisation. All models were adjusted for age (years), smoking status (current, former, never), race (White American, Black American, Other) and physical activity (not active, moderately active, active, highly active). Appendicular lean mass (ALM) was additionally adjusted for height and appendicular fat mass (AFM). ALM adjusted for height (ALM_ht_), fat free mass as a percentage of body weight (FFM%), _ext_SPPB, height-adjusted hand grip strength (HGS_ht_), and height-adjusted knee extension concentric (KEC_ht_) and isometric (KEI_ht_) strength were additionally adjusted for body mass index (kg/m^2^). The number of participants included in the full analysis (black) can be found in **Supplementary** **Tables 4 to 7**. The number of women included in the sensitivity analysis (grey) was: ALM, ALM_ht_ and FFM%: n = 615; _ext_SPPB: n = 620; HGS_ht_: n = 673; KEC_ht_: n = 503; KEI_ht_: n = 498. The number of men included in the sensitivity analysis (grey) was: ALM: n = 543; ALM_ht_ and FFM%: n = 542; _ext_SPPB: n = 537; HGS_ht_: n = 606; KEC_ht_ and KEI_ht_: n = 421. * p < 0.05, ** p < 0.01, *** p < 0.001.
